# Molecular Imaging of Vulnerable Atherosclerotic Plaques in Animal Models

**DOI:** 10.3390/ijms17091511

**Published:** 2016-09-09

**Authors:** Sara Gargiulo, Matteo Gramanzini, Marcello Mancini

**Affiliations:** Institute of Biostructure and Bioimaging, National Research Council, Via T. De Amicis 95, 80145 Naples, Italy; sara.gargiulo@ibb.cnr.it (S.G.); matteo.gramanzini@ibb.cnr.it (M.G.)

**Keywords:** atherosclerosis, vulnerable plaques, animal models, genetically modified mice, molecular imaging

## Abstract

Atherosclerosis is characterized by intimal plaques of the arterial vessels that develop slowly and, in some cases, may undergo spontaneous rupture with subsequent heart attack or stroke. Currently, noninvasive diagnostic tools are inadequate to screen atherosclerotic lesions at high risk of acute complications. Therefore, the attention of the scientific community has been focused on the use of molecular imaging for identifying vulnerable plaques. Genetically engineered murine models such as ApoE^−/−^ and ApoE^−/−^Fbn1C1039G^+/−^ mice have been shown to be useful for testing new probes targeting biomarkers of relevant molecular processes for the characterization of vulnerable plaques, such as vascular endothelial growth factor receptor (VEGFR)-1, VEGFR-2, intercellular adhesion molecule (ICAM)-1, P-selectin, and integrins, and for the potential development of translational tools to identify high-risk patients who could benefit from early therapeutic interventions. This review summarizes the main animal models of vulnerable plaques, with an emphasis on genetically altered mice, and the state-of-the-art preclinical molecular imaging strategies.

## 1. Introduction

Atherosclerosis is the primary cause of myocardial ischemia, stroke and lower limb amputation. It is a chronic disease that occurs predominantly at sites of disturbed laminar flow (arterial branches and bifurcations), which are characterized by the focal subendothelial accumulation of apolipoprotein B–containing lipoproteins and inflammatory cells (monocytes, macrophages, T cells, and mast cells) on injured intima, leading to the thickening of the arterial wall and formation of atherosclerotic plaques. The majority of atherosclerotic plaques remain clinically silent [1], but, in some cases, a plaque can become symptomatic via two mechanisms: (a) a gradual reduction of the vessel lumen leading to reduced blood flow, with the appearance of ischemic symptoms in conditions of high oxygen demand (stable angina pectoris, intermittent claudication); (b) erosion and rupture of the fibrous cap of the plaque with complete thrombosis of the lumen and/or embolization of the distal territory (heart attack or stroke) [2]. The broadest definition of a vulnerable plaque was proposed from a consensus document of the American Heart Association (AHA) in 2003: “The term ‘vulnerable plaque’ defines plaques susceptible to complications, and identifies all thrombosis-prone plaques and ones with a high probability of undergoing rapid progression, thus becoming culprit plaques” [3]. The term “vulnerable plaque” refers to often modestly stenotic lesions with a specific morphological presentation [4]: a large core of lipid deposits, necrotic cell debris and macrophages producing matrix-degrading enzymes such as metalloproteinases, with thinning of the fibrous cap.

These lesions are prone to rupture and may cause the above clinical complications [5]. The mechanisms underlying the transition of stable plaques to clinically significant lesions are currently not fully clear, but they involve a complex interplay of several biological processes, including inflammation, matrix remodeling, angiogenesis and apoptosis.

The present clinical and diagnostic tools are inadequate for the early identification of atherosclerosis and lesions that have a high probability to determine an acute event. Using a predictive model of risk assessment such as the Framingham risk score, it is impossible to accurately identify patients who will develop events and who would require immediate and aggressive therapeutic intervention. Therefore, the attention of the scientific community has focused on the development of new tools that provide higher sensitivity and specificity for the detection of vulnerable plaques. Molecular imaging can reveal specific biological pathways or cellular processes for a better understanding of the molecular events responsible for plaque destabilization. Moreover, the identification of novel imaging biomarkers may aid in risk stratification, with the potential to optimize preventive interventions that would reduce disease progression and cardiovascular events.

In vivo studies with experimental systems are essential to gain detailed insight into the molecular mechanisms of atherosclerotic plaque vulnerability and to develop better diagnostic and therapeutic tools. Animal models may enable us to study noninvasive imaging strategies to improve the ability to predict future risks of plaque rupture and to reduce the incidence of acute events. Although a variety of small and large animal models have been used for research on atherosclerosis, no model is currently considered ideal, and each has its own advantages and limitations with respect to the study of atherogenesis and modeling of the vulnerable plaque [6].

Until recently, useful large animal models have included nonhuman primates, pigs, and rabbits. Lesions in primates [7,8,9,10,11] and pigs [12,13,14] most closely resemble human lesions morphologically at all stages of the disease, and therefore they would be ideal models. In particular, the pig has a human-like lipoprotein profile and develops lesions in the coronary arteries [15,16], and porcine models of familial hypercholesterolemia have been shown to develop complex atherosclerotic lesions [17].

Rabbits have also been extensively used in atherosclerosis research, and some strains that exhibit familial hypercholesterolemia [18,19], such as the well-established Watanabe rabbit [20], develop coronary atherosclerosis. These models have certainly provided invaluable insight for defining the primary cellular events in the initiation and development of atherosclerotic lesions. Nevertheless, larger animals are more expensive to purchase, feed, handle and house in conditions appropriate to modern animal husbandry. Moreover, overall, large animals do not lend themselves to the ease and types of genetic manipulation that are possible in other species, such as mice [21]. At present, spontaneous plaque rupture in these models occurs only sporadically, after a long period of time, or depends on an additional trigger such as mechanical injury [22,23,24] or pharmacological treatment [25]. Additionally, the slow progression of complex atherosclerotic lesions, the long generation time, and ethical issues hinder the use of these species in preclinical molecular imaging research [26,27,28,29,30,31].

Although not all aspects are comparable to humans, the laboratory mouse (*Mus musculus*) is currently the most favored species to better understand the biology and pathology of atherosclerosis. The main advantages of this model include a well-known genetic background, a proportion of approximately 80% of genes in homology with humans [32], ease of breeding and low cost of maintenance. Moreover, the small size of mice facilitates the use of representative samples, which is a relevant issue for studying atherosclerotic lesions characterized by a large inherent variability.

In particular, murine models are recommended due to the relatively short time frame in which they develop atherosclerosis and due to their relative ease of genetic manipulation. Among these animal models, the genetically altered apolipoprotein-E–knockout (ApoE^−/−^) mice have been used extensively for the following reasons: they develop spontaneous atherosclerosis; the rate of atherogenesis can be notably accelerated by feeding the mice a high-fat, western-type diet (WTD); they exhibit various phases of the disease, including the early stage of fatty streaks, the accumulation of foam cells, and the development of a fibrous cap [33,34]; and they exhibit advanced stages of the disease characterized by relevant complications such as fissures, hemorrhage, plaque rupture and thrombosis [35].

Furthermore, depending on the experimental design, atherosclerotic lesions in this model may resemble the stable or unstable atherosclerotic lesions of humans [36,37,38]. Finally, ApoE^−/−^ mice have also been used to generate other relevant mouse models of atherosclerosis through breeding strategies [39,40,41]. Recently, a new genetically altered murine model of vulnerable plaques was established by crossbreeding ApoE^−/−^ mice with mice carrying a heterozygous mutation (C1039^+/−^) in the fibrillin-1 (Fbn1) gene [42]. Fbn1 is the major structural component of the extracellular microfibrils in the vessel wall, and the heterozygous C1039^+/−^ mutation led to fragmentation of the elastic fibers. Furthermore, it has been reported that ApoE^−/−^Fbn1C1039G^+/−^ mice seem to be associated with a more unstable plaque phenotype compared to ApoE^−/−^ mice because they develop acute plaque rupture, with subsequent myocardial infarction, neurological symptoms (head tilt, disorientation and motor disturbances), and sudden death. For these features, at the moment, transgenic ApoE^−/−^ mice are successfully being used as a model of stable atherosclerotic plaques, whereas ApoE^−/−^Fbn1C1039G^+/−^ mice could be considered to be a model for the study of the mechanisms of plaque rupture and for investigating theranostic tools.

Although the small size of the mouse could complicate the analysis of atherosclerotic vessels, recent technological advances in the field of high-resolution preclinical imaging such as magnetic resonance imaging (MRI) and contrast-enhanced ultrasound (CEUS) allow for the quantification of parameters of interest with high accuracy to monitor disease progression and may reduce the number of animals needed for research.

Both ApoE^−/−^ and ApoE^−/−^Fbn1C1039G^+/−^ mice have been shown to be useful tools for testing new imaging probes targeted for molecular markers of relevant biological processes such as VEGFR-1, VEGFR-2, ICAM-1, P-selectin and integrins. In this way, it is possible to noninvasively monitor the molecular pathways involved in the development of atherosclerotic plaques in vivo in these mice for comparison with those molecular pathways currently highlighted in humans, in a manner that is complementary to the use of molecular and cellular biology.

Very recently, the ApoE^−/−^Fbn1C1039G^+/−^ mouse model has been studied in vivo using gold nanoparticle–enhanced micro-computed tomography (microCT) to evaluate macrophage infiltration in atherosclerotic plaques at the level of the carotid bifurcation, with ex-vivo validation [43]. Genetically modified murine models such as ApoE^−/−^ and ApoE^−/−^Fbn1C1039G^+/−^ could offer promising avenues for testing innovative molecular imaging strategies as potential translational tools able to identify high-risk patients who could benefit from early therapeutic interventions.

## 2. Murine Models of Atherosclerosis and Molecular Imaging Targets

### 2.1. Murine Models of Atherosclerosis and Vulnerable Plaques

The study of atherosclerosis progression in humans is hindered by the complexity and chronicity of the disease process, by the difficulty to longitudinally monitor the changes of the plaques in an individual patient and by deficiency of the noninvasive detection modalities that provide limited information on the composition of the lesions.

The investigation of pathological changes in the arteries of humans is restricted to studies in the cross-sections of autoptic or surgical samples. Therefore, there has been a reliance on animal models, and, since 1992, the mouse has become an excellent system for the study of atherosclerosis, progressively replacing the use of large animals [44,45], in particular due to the ability to easily over- or under-express specific genes in this species. A number of recent reviews have extensively discussed the various mouse models of atherosclerosis available [46,47,48,49].

This review summarizes the relevant characteristics of the above experimental systems, with an emphasis on the currently available mouse models of vulnerable atherosclerotic plaques, comparing the pattern of their lesions to those in humans.

#### 2.1.1. Murine Models of Atherosclerosis: General Considerations

Unlike humans, in general, mice have high levels of antiatherosclerotic high-density lipoprotein (HDL) and low levels of proatherogenic low-density lipoprotein (LDL) and very low-density lipoprotein (VLDL) [46,47,48,49]. However, mice with specific genetic backgrounds can develop atherosclerosis on particular diets that promote an increase in the VLDL fraction, and the inbred strain C57BL/6 has been shown to be the most susceptible [46,47,48,49,50]. The earliest mouse model of atherosclerosis was induced in C57BL/6 mice with a special diet containing 30% fat, 5% cholesterol, and 2% cholic acid. However, this diet induced weight loss and often led to respiratory infections [46,47,48,49]. Therefore, subsequent studies have predominantly used C57BL/6 mice fed a diet supplemented with 15% fat, 1.25% cholesterol, and 0.5% cholic acid, commonly referred to as the “Paigen diet” [50].

Although this last model has been widely used in the study of atherosclerosis, unfortunately, the type of lesions did not resemble those in humans. In fact, the atherosclerotic plaques, which developed in these mice at four to five months of age, were very small (on the order of 200 to 1000 square microns) and were limited to the aortic root when the mice were fed the “Paigen diet” for periods of 14 weeks to nine months. Moreover, atherosclerotic lesions in this model were largely limited to the fatty streak stage, usually consisting mainly of macrophage foam cells and few smooth muscle cells (SMCs), and they did not progress to intermediate stages as in humans. Finally, the “Paigen diet” appeared to be unphysiological with regard to its extremely high cholesterol content and the presence of cholic acid, and it exhibited inflammatory properties [51].

To overcome these limitations, wild-type C57BL/6 mice are no longer used, and the current murine models for atherosclerosis have been primarily developed by genetic manipulations of and backcrossing onto C57BL/6, resulting in perturbations of the lipoprotein metabolism through adequate dietary modification or endogenous hyperlipidemia.

These genetically modified strains have the advantage of exhibiting extensive lesions at various stages of the disease, phenotypes of stable and vulnerable plaques and relevant complications such as plaque rupture and thrombosis. Although the current mouse models more closely represent human atherosclerosis, the choice should be based on the investigator’s specific needs as each model has distinctive features and some limitations. Several studies have demonstrated the feasibility of these models. In contemporary research on atherosclerosis, the most extensively characterized strains are those deficient in apolipoprotein E (ApoE^−/−^) and low-density lipoprotein (LDL^−/−^) receptors [52,53,54]. The introduction of these gene-targeted mice has revolutionized the study of atherogenic processes and has ensured that the mouse quickly became the most popular mammalian model of atherosclerosis to date. A specific allele is deleted, permitting the precise definition of a protein’s activities. These two murine models have several advantages as follows: they are readily commercially available, they breed well, and their lesions have been documented in detail within a well-defined time frame and appear morphologically comparable to those found in humans [33,34,55].

In ApoE^−/−^ mice, the normal gene coding for apolipoprotein E is replaced by a mutated gene that does not produce this molecule, leading to elevation of cholesterol-enriched chylomicrons and very low-density lipoprotein (VLDL)-sized particles. The first mouse model with a switched-off gene for apolipoprotein E was generated almost contemporaneously in two laboratories in the United States [56,57], and shortly thereafter this model was described as “the best animal model of atherosclerosis” available at that time [58]. In contrast to previous animal models, this mouse is able to develop atherosclerosis spontaneously [59], and it is possible to accelerate the development of the disease using a high-fat diet similar in composition to an average American diet (21% fat, 0.15% cholesterol), called a “western-type” diet [55]. Gender- and age-related differences in cardiovascular aging have been demonstrated in this genetically modified mouse, since hypercholesterolemia and male gender additively aggravate the entity of lipid deposition and vascular senescence in ApoE^−/−^ mice of advanced age [60].

In LDL^−/−^ mice, the deletion of the LDL receptor mimics the homozygous form of genetic hypercholesterolemia found in humans; however, these mice exhibit only modest hyperlipidemia when fed a normal diet, whereas they are susceptible to the formation of atherosclerotic lesions when fed a high-fat diet.

**Figure 1:**
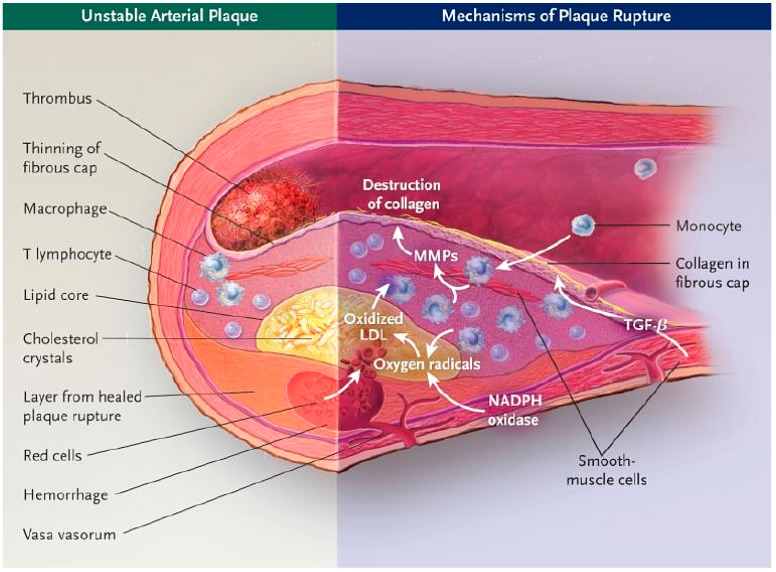
Pathogenesis of atherosclerosis and the mechanisms of plaque rupture. Unstable plaque has a thin fibrous cap, may be thrombotic, and is characterized by many inflammatory cells and a large lipid core. Accumulation of macrophages and T lymphocytes in plaques leads to the release of matrix metalloproteinases (MMPs), which digest collagen and cause thinning of the fibrous cap. The necrotic lipid core grows as a result of the accumulation of lipids in the extracellular matrix, the death of lipid-laden macrophages, and perhaps the accumulation of erythrocyte membranes after intraplaque hemorrhage from the vasa vasorum. Oxygen radicals, generated mainly from NADPH oxidase and inflammatory cells, oxidize low-density lipoproteins (LDL) and cause necrosis of cells. (Reprinted from Reference [61]. Copyright with permission from © 2003, Massachusetts Medical Society.)

Because this review is intended to highlight the use of murine models to study the process of atherogenesis and, in particular, vulnerable plaques, a brief description of the morphological features of the six stages of atherosclerotic lesions in ApoE^−/−^ and LDL^−/−^ mice, based on the human scale of AHA, is provided (as shown in Figure 1) [48,61].

Stage I lesions consist of the focal accumulation of lipids with slight intimal thickening. The progression to stage II involves the infiltration of lymphocytes and macrophages, with the latter referred to as “foam cells”, due to the intracytoplasmic lipidic inclusions. This type of lesion, defined as a “fatty streak”, appears as a yellowish stripe on the intimal surface and is a common feature in the aortas of both ApoE^−/−^ and LDL^−/−^ mice. The accumulation of extracellular lipids is detected in lesions that undergo progression to stage III. The first lesion considered advanced by histological criteria is stage IV, presenting a further increase and convergence of extracellular lipids to form a “lipid core” covered with foam cells. Afterwards, a “cap” of fibrous tissue progressively develops, and thus the stage V lesions are defined as “fibrous plaques”. Macrophage-derived foam cells and lymphocytes are generally found densely concentrated along the periphery of the lipid core of both stage IV and V lesions [62,63]. Stage IV and V lesions are capable of developing fissures, hematomas, and/or thrombi and, for this reason, are clinically relevant in terms of morbidity and mortality in humans. Finally, plaques called “complicated” correspond to stage VI, because these lesions are characterized by ulcerations, hematomas, hemorrhages or thrombi, and may generate acute events.

Although both mouse models develop atherosclerotic plaques at the level of the aorta after being fed a high-fat diet which closely resemble the morphological features of human atherosclerotic plaques, ApoE^−/−^ mice develop more extended lesions in a shorter time frame [55,57,64].

Moreover, the atherosclerotic plaques have been described in ApoE^−/−^ mice not only in the aortic root but throughout the entire aorta (descending thoracic and lower abdominal aorta, aortic bifurcation and common iliac vascular tracts) and its principal branches (brachiocephalic and right common carotid arteries). With a standard diet, fatty streaks were first observed in the proximal aortas of three-month-old ApoE^−/−^ mice, and foam cell lesions were seen at 10 weeks of age [55]. Intermediate lesions containing foam cells and SMCs appeared at 15 weeks, and fibrous plaques at 20 weeks of age. The western diet markedly increases plasmatic VLDL and chylomicrons [56,57] and accelerates the atherogenic process, resulting in the detection of fatty streaks by eight weeks of age and the appearance of complications such as intralesional calcifications [55,65]. In comparison, LDL^−/−^ mice fed a regular chow diet display high individual variability in lesion development, whereas those subjected to a high-fat diet develop atherosclerotic lesions specifically in the aortic root and innominate artery at approximately three months of age. Lesions extended to the thoracic aorta at six months and the abdominal aorta at nine months [66]. Moreover, plaques formed in LDL^−/−^ mice are representative only of the I–III phases, consisting predominantly of macrophage foam cells, while advanced lesions develop solely after prolonged exposure to a fat-enriched diet and do not progress beyond stage IV of the AHA-defined scale of human atherosclerosis [33,34,64]. Conversely, at 45–54 weeks of age in ApoE^−/−^ mice that are fed an atherogenic diet for a prolonged period of time (14 to 20 weeks), lesions classified as stage VI are observed, but only in the innominate artery [35,67], while they have never been documented in the aortic root or along the aortic tree [55,68].

In particular, lesions in the innominate artery may provide valuable insight because they more frequently exhibit complex features comparable to vulnerable plaques in humans, including an acellular necrotic core, erosion of the necrotic mass through to the lumen or intraplaque hemorrhage [35], and, although its small size provides some technical challenges, this site will likely become more popular with time. LDL^−/−^ mice have been found to be most suitable for studying the genetics of primary or secondary dyslipidemias, while ApoE^−/−^ mice have been established as the best murine model for characterizing the cytological and molecular aspects of stable and vulnerable plaques. Atherosclerotic plaques with visible defects of the fibrous cap and hemorrhage have been described in the innominate arteries of 37- to 59-week-old ApoE^−/−^ mice fed a high-fat diet for approximately two months [69] or in younger mice at eight to 16 weeks of age [70], but acute events have occurred rarely [71,72], or without sufficiently reliability, after prolonged feeding of a western diet (10 months) [70].

Some groups use the transgenic ApoE*Leiden mice, which are knock-in mice expressing the human apolipoprotein E3 isoform with low affinity for the LDL receptor. These mice are less hyperlipidemic than ApoE^−/−^ mice, spontaneously develop atherosclerosis when fed the atherogenic diet, and are suitable for exploring the mechanisms by which apolipoprotein E isoforms influence hepatic VLDL metabolism in atherogenesis [73].

#### 2.1.2. Murine Models of Vulnerable Atherosclerotic Plaques

The study of destabilized lesions appeared to be difficult and to require a long observation time in these murine models, thus stimulating the improvement of their efficiency and reproducibility regarding the salient features of spontaneous rupture of plaques in humans. Therefore, in the last decade, several strategies have been proposed in ApoE^−/−^ mice to generate models in which plaque rupture occurs in a reasonably short period of time that allows for the observation of the rupture of plaques and for testing diagnostic and therapeutic interventions. While a stable atheromatous plaque is most commonly covered with a fairly thick fibrous layer, protecting the lipid nucleus from contact with the blood, vulnerable plaques are characterized by a large lipid-rich core covered by a thin fibrous cap, with extensive macrophage infiltration, but very few SMCs [3,74]. These unstable plaques may undergo erosions, which is a loss of endothelium leading to thrombus formation [75] or rupture, defined as disruption of the fibrous cap accompanied by intrusion of erythrocytes into the lesion itself [67].

The first mouse models of destabilized plaques were mainly generated from the ApoE^−/−^ strain using microsurgical procedures, such as compressive injury using blunt forceps applied to atheromatous lesions in the abdominal aorta [76] or disruption of the fibrous cap in advanced plaques using a needle [77]. These experimental systems have primarily been a valuable model for studying post-rupture thrombosis, but they have not provided a tool for investigating the spontaneous rupture of atherosclerotic plaques.

Further invasive approaches to develop unstable plaques were aimed at generating hemodynamic changes both in ApoE^−/−^ mice fed a high-fat diet, for example by placing a perivascular device [36,78] or introducing a tandem stenosis in the carotid arteries [38], and in ApoE^−/−^ mice fed a standard diet, using two different interventions (ligation plus cuff positioning) on common carotid arteries [79,80] or using ligation of external and internal carotid arteries and part of the renal artery to induce hypertension [81]. A model of cuff-induced thrombosis, produced by placing a silastic collar around the left common carotid artery in ApoE^−/−^ mice fed a normal chow, was used to evaluate the efficacy of intravenous administration of mononuclear cells, demonstrating the attenuation of the progression of atherosclerosis, reducing endothelial dysfunction, formation of thrombi, oxidative stress and apoptosis [82]. However, the induced biomechanical alterations might not reflect the pathogenesis of spontaneous plaque rupture in humans and could call into question the interpretations of the experimental results in these models [83,84]; additionally, the invasive nature of the procedures could represent a limitation for both ethical and practical reasons. The incidence of plaque rupture in ApoE^−/−^ mice with collar-induced lesions in carotid arteries was also increased by adenovirus-mediated over-expression of p53, a pro-apoptotic stimulus for intralesional SMCs producing a cap thinning, combined with phenylephrine injection, a defined hemodynamic stimulus [85]. Nevertheless, the requirements for both survival surgery and carotid viral transfection may not be readily available for high-throughput experimental protocols in all laboratories.

Different biological approaches have also been described in the literature on the ApoE^−/−^ strain to generate mouse models reminiscent of human vulnerable plaques. Such interventions have included: alterations of transforming growth factor-β signaling, which produced thinning of fibrous caps, large lipid cores, intraplaque hemorrhage and disruption of the endothelium [86,87]; deletion or inhibition of Gas6, a platelet-response amplifier, which induced large intraplaque hemorrhages in the absence of fibrous cap fissuring [88,89]; and a null mutation for high-density lipoprotein receptor SR-BI, resulting in severe occlusive coronary atherosclerotic lesions leading to myocardial infarctions [90]. Gough et al. [91] induced molecular weakening of the fibrous cap by transfection of ApoE^−/−^ mice with hematopoietic stem cells over-expressing matrix metalloproteinase (MMP) 9. Fundamentally, degradation of collagen reduces the strength of the fibrous cap and, therefore, enhances the risk of its rupture. Another study combined collar placement on the carotid artery and short-term lipopolysaccharide (LPS) administration together with phenylephrine treatment and cold [92]. LPS leads to an increase in Th17 cells, which, through IL17, induces apoptosis of SMCs, potentially decreasing collagen content in the plaque, while phenylephrine, a vasoconstrictor, leads to increased blood pressure. Also, in these models, the complex experimental methods and specialized expertise required to induce plaque rupture may represent a limitation for their extensive use.

Recent evidence both in humans and mouse models [93] has indicated that angiotensin II (Ang II) plays a pivotal role in atherogenesis. The pro-atherosclerotic actions of Ang II are mediated by the AT1 receptor, which is expressed in a variety of organs, blood vessels, and bone marrow–derived cells, such as macrophages and T cells [94,95]. Therefore, subcutaneous and chronic infusion of Ang II has recently emerged as a practical and reproducible stimulus to spontaneously induce the development of vulnerable plaques in ApoE^−/−^ mice, both in the aorta and in its branches, especially the innominate artery [96,97,98,99], even after discontinuation [37]. Ang II likely promotes the destabilization of plaques through multifactorial mechanisms, including hypertension, chemotaxis of monocytes, activation of macrophages, altered sympathetic regulation of vasal tone, altered secretion of aldosterone and prostaglandin, generation of reactive oxygen species and over-expression of monocyte chemoattractant protein-1 (MCP-1) in vascular SMCs. Overall, these events are responsible for intralesional neovascularization, hemorrhages, inflammation and remodeling of plaque [100,101]. Occlusive vascular events are not described in this model, most likely due to differences in clotting mechanisms between mice and humans, but, in reality, not all thrombotic events lead to clinically relevant occlusive events, and thus the model lends itself easily to the study of the process of plaque destabilization in various stages of evolution. Ang II infusion may be implemented in mice through the use of different models of osmotic pumps, subcutaneously implanted, which provide a constant release of Ang II over time and differ in dosage and duration of treatment. Several protocols are described in the literature, and the most accredited involves the administration of Ang II at a dose of 0.7 mg/kg/day for four to eight weeks, four weeks from the start of the high-fat diet at eight weeks of age [97].

Diabetes is well recognized as a prevalent risk factor for atherosclerosis, and cardiovascular incidents as a consequence of plaque rupture are significantly increased in diabetic patients [102,103]. Diabetes triggers endothelial cell dysfunction and lipid abnormalities, promoting the expression of adhesion molecules, chemokines and other proinflammatory mediators. Moreover, atherosclerotic plaques in diabetics exhibit reduced collagen synthesis and increased breakdown of collagen, which predispose these patients to plaque rupture and a higher risk of thrombus formation [104]. In fact, hyperglycemia leads to the formation of oxidized glycated LDLs, which in turn induce vascular SMC apoptosis and reduced collagen content within plaques and may enhance thrombogenesis by activating platelets and reducing the production of endogenous platelet inhibitors [105].

This evidence has highlighted the importance of studying the mechanistic link between diabetes and atherosclerosis. In the past decade, several diabetic atherosclerosis mouse models have been established, and, despite their limitations, they currently have a leading role for these purposes. Convenient models are nontransgenic or transgenic mice. One of the first mouse models of diabetic atherosclerosis was the diet-induced obese (DIO) C57Bl/6J mouse, but only 40% of these mice developed small lipid deposits in the aortic sinus after 14 weeks of a diabetogenic diet (DD, 35.5% energy from fat); therefore, this appeared to be a poor model for the study of atherosclerotic complications in diabetes [106]. The genetically modified mouse models ApoE^−/−^ (King 2010; Canizzo 2012) [107,108] and LDL^−/−^ [109,110] also failed as pure diet-induced diabetic atherosclerosis models, because they have exhibited inconsistent effects on blood glucose or insulin levels or on the development of insulin resistance, and the variable increment of plaques would mainly be related to hyperlipidemia rather than to alterations in glucose metabolism.

The most common mouse models that closely resemble human diabetic atherosclerosis have been produced using two different approaches, alternative or complementary to the manipulation of diet: chemical toxins or additional genetic manipulation in dyslipidemic atherosclerosis-prone strains. Streptozotocin (STZ) is a toxic glucose analogue that accumulates in pancreatic β-cells and causes cell death leading to type 1 diabetes (T1D) with hyperglycemia. Induction of diabetes with STZ has been performed in different atherosclerosis models, and especially in ApoE^−/−^ mice [111]. Diabetic ApoE^−/−^ mice exhibited a significant increase in plaque area at the level of the aortic arch and the proximal aorta compared to controls [112,113,114], but it is difficult to discriminate whether the accelerated atherosclerosis is due to hyperglycemia or dyslipidemia. However, deranged carbohydrate and lipid metabolism is a common feature in human diabetic patients, thus highlighting the relevance of such available murine models.

Interestingly, Veerman et al. [115] found that STZ-induced hyperglycemia in ApoE^−/−^ mice was associated with low density of the vasa vasorum and under-expression of VEGF-R2. The use of STZ has the advantage of a standardized protocol, onset and development of diabetes, but does not fully replicate T1D as it usually does not induce ketosis and does not necessitate insulin therapy. Moreover, although it has been reported that diabetes accelerates atherosclerosis in these mouse models, a potential problem with STZ treatment is the toxic effect in many organs. The issue of high plasmatic levels of lipids in the widely used models of atherosclerosis has been overcome by crossing mice expressing human aldose reductase (hAR) with LDLR^−/−^. In fact, hAR-LDLR mice are a model of accelerated atherosclerosis that does not exhibit hyperlipidemia [116]. This mouse model has confirmed that AR expression influences atherosclerosis development and that hyperglycemia alone, in conjunction with a particular genetic background, may accelerate the progression of this disease.

Recently, genetically modified models of diabetic atherosclerosis have been developed using Ins2AKITA (or Akita) mice on ApoE^−/−^ or LDLR^−/−^ backgrounds. Akita mice have a mutation in the insulin 2 gene (Cys96Tyr), leading to a misfolding of the proinsulin 2 protein, beta cell apoptosis, and T1D. Akita mice crossbred to ApoE^−/−^ [117] and LDLR^−/−^ [118,119] have a three-fold increase in non-HDL cholesterol and triglyceride levels and an accumulation of inflammatory cells in plaques. Wang et al. [120] induced T1D with STZ in ApoE^−/−^/LDL^−/−^ mice fed a WTD and found that they displayed a more unstable phenotype of atherosclerotic plaques than nondiabetic controls fed a normal diet. They also found that the amelioration of insulin resistance through the silencing of Tribbles homolog 3 (TRIB3), an inhibitor of the Akt phosphorylation proatherogenic pathway, was able to improve plaque stability. Wu et al. [121] crossed ApoE^−/−^ mice with db/db mice to generate a type 2 diabetic (T2D) atherosclerosis model. ApoE^−/−^db/db mice exhibited significantly accelerated atherosclerosis in the aorta, and Wendt et al. [122] found that ApoE^−/−^db/db mice display enhanced expression of VCAM-1 and MMP-9 in the aorta.

Arterial calcification has emerged as a significant marker of advanced atherosclerosis, and calcium score is a well-established clinical predictor of cardiovascular risk [123,124]. However, the link between intimal calcification and plaque vulnerability is still controversial, and several factors such as morphology, size and location, in relation to the amount of calcium deposits, may affect lesion stability [125]. It is reported that large calcifications, easily detected with coronary computed tomography, do not appear to increase plaque vulnerability [126], whereas small calcifications within the fibrous cap can lead to biomechanical stress that can cause plaque rupture. Therefore, microcalcifications are recognized to play a role in destabilizing atherosclerotic plaques [127,128], and murine models of atherosclerotic calcification have been developed to better understand its pathological role and to test the effects of new therapies on this prognostic factor.

Several inbred mouse strains, including C57BL/6, Balb/C, C3H/HeJ, DBA/2J, and SM/J, develop spontaneous artery wall calcification with different occurrence, particularly when fed a high-fat/high-cholesterol diet [129,130], indicating genetic regulation of this phenomenon. Vascular calcification occurs spontaneously in genetically modified ApoE^−/−^ mice, which were shown to have marked cartilaginous metaplasia in the brachiocephalic artery [35,131], and is accentuated by osteopontin deficiency [132,133]. Debernardi et al. [134] investigated the presence of microcalcifications in mechanical-induced atherosclerotic lesions of the carotid artery in ApoE^−/−^ mice, and they proposed the use of this mouse model to investigate the pathophysiological significance of accumulation of elements such as calcium, iron and zinc during the atherosclerotic process. Moreover, Towler et al. [135] demonstrated that vascular calcification occurs in response to a high-fat, diabetogenic diet in LDL^−/−^ mice.

Unraveling the mechanisms underlying cardiovascular calcification may be an important step towards future strategies. Interestingly, research has demonstrated that dyslipidemia and inflammation may link reduced bone mineral density and vascular calcification: it has been reported that patients with low bone density have more pronounced atherosclerotic plaque calcification [136,137], and different preclinical studies have suggested that hyperlipidemia promotes both arterial calcification and bone loss in the ApoE^−/−^ model [138,139,140]. Very recently, Li et al. [141] tested the effects of simvastatin treatment in the ApoE^−/−^ model and showed a significant reduction of microcalcification in atherosclerotic plaques.

**Figure 2:**
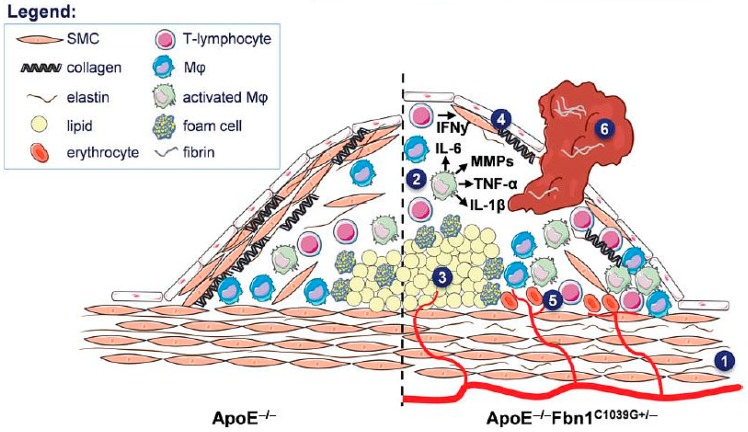
Mechanisms leading to the formation of vulnerable plaques, plaque rupture, myocardial infarction, stroke, and sudden death in ApoE^−/−^Fbn1C1039G^+/−^ mice. In ApoE^−/−^Fbn1C1039G^+/−^ mice, elastin fragmentation (**1**) and arterial stiffness lead to the development of large plaques with a highly unstable phenotype, characterized by enhanced inflammation (**2**), large necrotic cores (NC) (**3**) and a thin fibrous cap (FC) (**4**). Additionally, in the brachiocephalic and carotid arteries intraplaque neovascularization and hemorrhage (IPH) are abundantly present (**5**). Due to the elevated pulse pressure and extensive aortic dilatation (especially in the ascending aorta), the mechanical stress on plaques is increased, leading to rupture and subsequent thrombus formation (**6**). Plaque rupture with thrombosis as well as hypoperfusion of the heart and brain most likely result in myocardial infarction, stroke, and eventually sudden death. (Reprinted from Reference [42]. Copyright with permission from © 2014, Oxford University Press on behalf of the European Society of Cardiology.)

A recent model has investigated the effect of impaired fibrillin-1 function on atherosclerosis in ApoE^−/−^C1039G^+/−^ double-knockout mice on a western-type diet. Mice with a heterozygous mutation in the fibrillin-1 gene (Fbn1C1039G+/−) develop fragmentation of the elastic fibers and were crossbred with ApoE^−/−^ mice to generate reduced levels of elastin in their vessel walls and, consequently, stiffer vessels [142]. The loss of elastin would result in exposure of the fibrous cap of the atherosclerotic plaques to increased biomechanical stress. Indeed, after 10–20 weeks of high-fat diet, these mice developed larger plaques with features of vulnerability, such as a decrease in collagen content, increased apoptosis of SMCs, large necrotic cores, an increase in macrophage infiltration, and numerous buried caps, not only at the level of the aortic valves but also in the brachiocephalic artery and in different tracts of the aorta. Furthermore, after being fed a western-type diet for up to 35 weeks, a large number of the mice showed coronary plaques fissured with thrombi [42], and spontaneous acute plaque disruption was reported [143] and described in a large percentage of the mice in combination with symptoms of myocardial infarction, stroke or sudden death [42,142,144] (as shown in Figure 2).

The level of macrophage infiltration was highlighted as one indicator of plaque vulnerability in the ApoE^−/−^C1039G^+/−^ mouse model and was quantified in vivo by gold nanoparticle–enhanced microCT at the level of the common, external, and internal carotid arteries, demonstrating a more rapid development and a larger extent of plaques in the ApoE^−/−^C1039G^+/−^ mice compared to the ApoE^−/−^ mice [43]. Very recently, a new mouse model was introduced with an inducible adenovirus-mediated gain-of-function mutation, D374Y, in the proprotein convertase subtilisin/kexin type 9 (PCSK9) gene, which is another important factor that regulates lipid homeostasis like the ApoE and LDL proteins [145]. This PCSK9^DY^ mutation in ApoE^−/−^ mice mimics a genetic condition of hypercholesterolemia in patients, and it showed a synergistic effect in combination with ApoE deficiency, resulting in a strong increase in serum low-density lipoprotein, accelerated plaque growth and doubling of lesion size compared to wild-type ApoE^−/−^ mice. Therefore, intravenous administration of an adenoviral vector for stable transfection of the PCSK9^DY^ mutated gene in ApoE^−/−^ mice has been described as a promising approach to study the development of advanced atherosclerotic plaques related to the interaction of different genetic mutations, without time-consuming backcrosses, and further long-term studies to well characterize this model are expected to be published [146].

Finally, as not the least-relevant aspect, it is known that although some features of plaque instability have been reproduced in genetically modified mouse models, atherothrombosis induction is difficult. In a recent work by Liu et al. [147], murine prothrombin was over-expressed via adenovirus-mediated gene transfer in an ApoE^−/−^ mouse model of plaque destabilization [148]. This approach produces features of plaques that are found in human coronary arteries including fibrous cap disruption, plaque hemorrhage and luminal thrombosis in 70% of the animals, suggesting that blood coagulation is critical to consistently reproduce atherothrombosis in the mouse model.

The major mouse models of atherosclerosis are briefly summarized in Table 1.

### 2.2. Molecular Biomarkers of Atherosclerotic Plaques in Humans and Murine Models

**Figure 3:**
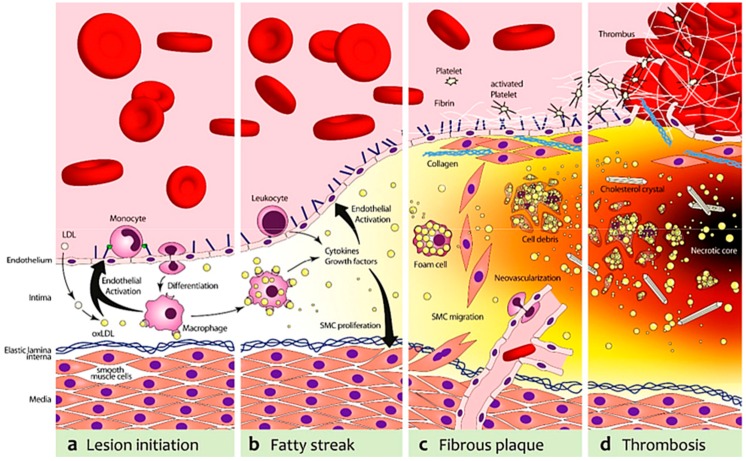
Schematic depiction of representative targets for molecular imaging of atherosclerosis and plaque vulnerability. (**a**) In the first stage, low-density lipoprotein cholesterol (LDL) is deposited in the endothelium and undergoes oxidative modification, resulting in oxidized LDL (oxLDL). OxLDL stimulates endothelial cells to express adhesion molecules (vascular cell adhesion molecule-1 (VCAM-1), P-Selectin and various chemokines, e.g., Monocyte Chemoattractant Protein-1 (MCP-1) and Interleukin 8 (IL-8). This leads to a recruitment of monocytes, which transmigrate into the intima and differentiate to pro-atherogenic macrophages; (**b**) Macrophages harvest residual oxLDL via their scavenger receptors and add to the endothelial activation and, subsequently, leukocyte recruitment with the secretion of Tumor Necrosis Factor α (TNF-α) and IL-6; (**c**) The increasing plaque volume promotes neovascularization. Proliferating smooth muscle cells (SMCs) stabilize the nascent fibrous plaque. With deposition of fibrin and activated platelets on the dysfunctional endothelium that expresses tissue factor (TF) and von Willebrand factor (vWF), a pro-thrombotic milieu is formed; (**d**) Foam cells can undergo apoptosis and release cell-debris and lipids, which will result in the formation of a necrotic core. In addition, proteases secreted from foam cells can destabilize the plaque. This can lead to plaque rupture, in which the case of extracellular matrix molecules (e.g., collagens, elastin, TF, vWF) catalyze thrombotic events. (Reprinted from Reference [149]. Copyright with permission from © 2015, MDPI, Basel, Switzerland, Creative Commons Attribution License CC BY 4.0.)

The characterization of molecular markers expressed both in humans and in murine models is relevant to promote the translation of novel noninvasive diagnostic and therapeutic approaches for vulnerable plaques from preclinical research to the clinic (as shown in Figure 3) [149].

Several works have mainly evaluated the major receptors expressed at the level of atherosclerotic plaques of ApoE^−/−^ mice both ex vivo and in vivo. Therefore, we further summarize the current status of the knowledge of the main molecular factors identified in patients and in the murine models of atherosclerosis currently available, with a focus on the most promising imaging targets of key processes implicated in plaque destabilization.

#### 2.2.1. Leukocyte Adhesion Receptors

Inflammation is recognized as a crucial factor for the development of atherosclerotic lesions and the progression of their vulnerability. In fact, macrophage-rich plaques appear to be more prone to rupture. Experimental evidence shows that in humans and animal models of atherosclerosis, one of the observed changes in the endothelium is an increased expression of leukocyte adhesion receptors, such as P-selectin, E-selectin, VCAM-1, and ICAM-1 [150,151], which allow monocytes to roll and adhere to the artery wall and then enter the subendothelial space. Recently, the expression of several molecules of leukocyte adhesion in human atherosclerotic plaques, obtained from autopsies or from the hearts of patients undergoing heart transplantation, has been reviewed [152]. VCAM-1, which binds the very late antigen-4 (VLA 4) on the surface of leukocytes, was found to be expressed by activated endothelial cells, macrophages and SMCs even in the early stages of the atherogenic process, playing a major role in the recruitment of inflammatory cells. However, variable levels of VCAM-1 have been reported on the endothelium in human atherosclerotic lesions. Davies et al. [153] found that VCAM-1 appears focally on endothelial cells covering fibrous or lipid-rich plaques, on intraplaque neovessels, and on SMCs and macrophages. O’Brien et al. [154] identified significant expression of VCAM-1 on SMCs in atherosclerotic coronary plaques of humans, while its presence on the luminal endothelium was low in both lesions and control segments but was prevalent in association with the intimal neovasculature.

Despite the diversity of such data, George et al. [155] indicated that VCAM-1 is a very interesting diagnostic and therapeutic target because it is expressed only on activated endothelial cells that line the surface of atherosclerotic plaques, while it does not appear to be significantly expressed on the endothelium in non-affected areas. Furthermore, preclinical studies have shown that blocking or knocking out this receptor significantly inhibits macrophage recruitment and the formation of plaques. Mice with hyperglycemia exhibit increased VCAM-1 expression, and similar findings have been obtained in isolated human endothelial cells exposed to elevated glucose in vitro [156]. The over-expression of ICAM-1 has been demonstrated not only on the endothelium but also on SMCs and macrophages of human atherosclerotic plaques [153,157,158].

An increase in the levels of the adhesive molecules VCAM-1 and ICAM-1 at sites with atheromatous changes was also observed in ApoE^−/−^ mice. Nakashima et al. [159] characterized VCAM-1 on the endothelium of ApoE^−/−^ mice by immunohistochemistry, highlighting its early expression at the level of vascular sites susceptible to the development of plaques and at the periphery of the lesions in advanced stages. Few endothelial cells with a weakly positive VCAM-1 signal were instead shown in the control subjects. They also identified ICAM-1 on the endothelium at the same vascular sites, but without statistically significant differences compared to the controls [159]. In fact, the expression of ICAM-1 has been reported to be constitutively high in the endothelium of cardiac vessels of murine species [160]. In human atherosclerotic lesions, a marked expression of P-selectin has been demonstrated on the endothelium overlying active atherosclerotic plaques, but not on the normal arterial endothelium or covering fibrous plaques [161].

The role of P-selectin in the spontaneous development of advanced atherosclerosis was also evaluated in ApoE^−/−^ mice. P-selectin appears to be a key adhesion receptor mediating the recruitment of monocytes/macrophages into the lesions and promoting advanced atherosclerosis in ApoE^−/−^ mice, with earlier and more advanced lesions in mice lacking ApoE alone in comparison with double-knockout ApoE/P-selectin mice [162]. In accordance with this, Ramos et al. [163] demonstrated that anti–P-selectin antibodies inhibit monocytes rolling on the endothelium of carotid arteries isolated from ApoE^−/−^ mice. Furthermore, some studies have highlighted that advanced atherosclerotic lesions are actively promoted in ApoE^−/−^ mice not only by endothelial P-selectin but also by platelet P-selectin [164,165]. In humans, the expression of E-selectin is limited to endothelial cells on the surface of fibrous and lipid-rich plaques [153], whereas in the aortas of normal chow-fed ApoE^−/−^ mice, E-selectin was not expressed on endothelial cells in regions predisposed to atherosclerosis or in early and advanced plaques.

#### 2.2.2. Indicators of Macrophage Infiltration

A large number of macrophages in atherosclerotic lesions is an indicator of a more unstable and rupture-prone phenotype [166,167]. In fact, these cells accumulate in plaques and phagocytize lipids, turning into foam cells, and release cytokines and growth factors, as well as metalloproteinases and reactive oxygen species that degrade the structures of plaques [168]. Therefore, the quantification of macrophages is a potential target for the identification of vulnerable plaques. Xu et al. [169] demonstrated the expression of Toll-like receptor 4 (TLR-4) in the aortic atherosclerotic lesions of ApoE^−/−^ mice and in human coronary artery autopsy specimens by immunohistochemistry. Activated macrophages also express the receptor for folate (FR β). Ayala-Lopez et al. [170] demonstrated high FR β expression on macrophages in aortic atherosclerotic lesions of ApoE^−/−^ mice both in vivo using nuclear medicine imaging and ex vivo using immunohistochemistry, while Müller et al. [171] showed increased FR β expression in human specimens obtained from carotid endarterectomy, which co-localized with the macrophage activation marker “Cluster of Differentiation 68” (CD68).

#### 2.2.3. Indicators of Angiogenesis

Histological examination of atherosclerotic plaques reveals a rich neovascularization. The microvessels most likely form in response to over-expressed angiogenic factors, including VEGF. Angiogenesis in plaques may favor their growth, a phenotype that is friable and prone to rupture and that leads to hemorrhages and thrombosis.

The expression of VEGF and its receptors (VEGFRs) by endothelial cells, macrophages and other cell types has been implicated in the development of atherosclerosis and vulnerable plaques, particularly in association with diabetes, both in humans [172,173,174,175,176] and in animal models [177,178]. An association between neovascularization and atherosclerosis has been confirmed by several studies, showing a correlation between the extent of atherosclerosis and plaque neovascularization in human pathological samples [179,180,181,182]. Enhanced VEGF/VEGFR signaling plays an important role in three critical processes leading to plaque vulnerability: stimulation of angiogenesis in plaques, recruitment of monocytes into plaques, and increasing permeability of plaque vasculature which leads to hemorrhage and inflammatory cell extravasation [174,175].

The strongest experimental evidence that angiogenesis plays a pathogenetic role in atherosclerosis was derived from studies in the ApoE^−/−^ mouse model. Moulton et al. [183,184] provided the first confirmation that the process of angiogenesis is involved in the progression of atherosclerosis, showing that neovascularization is seen in advanced atheromas of ApoE^−/−^ mice and that their development may be significantly reduced by specific endothelial inhibitors. However, the incidence of lesions with intimal vessels reported by Moulton et al. [183,184] in this mouse model was relatively low (13%); therefore, subsequent studies were performed in ApoE^−/−^ mice with streptozotocin-induced diabetes. Immunohistochemistry showed a significant increase of VEGFRs in aortic plaques, as well as the co-localization of VEGFR-1 primarily with the macrophage marker Mac3 and VEGFR-2 with the endothelial cell marker FVIII, highlighting their potential use in novel molecular imaging strategies [185]. Another potential marker for the targeting of angiogenesis in atherosclerotic lesions is α_v_β_3_ integrin, a cell surface glycoprotein receptor that is highly expressed by macrophages, medial and some intimal SMCs, and endothelial cells [186,187,188]. Expression of α_v_β_3_ integrin is found in the shoulder of advanced plaques and in the necrotic core of human atherosclerotic lesions [189]. Moreover, for in vivo imaging purposes, integrin α_v_β_3_ is the most extensively examined marker of angiogenesis [190,191].

#### 2.2.4. Other Potential Molecular Targets

Vulnerable plaques are characterized by the presence of apoptotic cell death and induction of atherothrombosis [5]. In advanced lesions, apoptosis of macrophages promotes thinning of the fibrous cap and the development of the necrotic core, a key factor in rendering plaques vulnerable to disruption and in the formation of luminal thrombi [192,193]. During apoptosis, phosphatidylserine (PS) is externalized on the cell membrane [194,195] and is recognized by the 35 kDa plasma protein Annexin V (A5) [196]. Therefore, A5 has been effectively used for various molecular imaging strategies in preclinical atherosclerosis models including ApoE^−/−^ mice [185,197,198], as well as in human cardiovascular disease [199,200]. The fibrous cap separates platelets and prothrombotic materials in the plaque. Rupture of this barrier leads to the exposure of a variety of intraplaque constituents to the circulation, initiating atherothrombosis which may cause stroke or myocardial infarction. Therefore, thrombus formation is an important aspect in the instability of atherosclerotic lesions, and molecules present on activated platelets such as glycoprotein IIb/IIIa [201] or the specific collagen receptor glycoprotein VI (GPVI) [202], selective targets of fibrins [203,204], clotting factors or components of the exposed subendothelial matrix [205,206] can represent an interesting strategy for assessing their vulnerability, also using various molecular imaging techniques.

### 2.3. Emerging Applications for Molecular Imaging in Murine Models of Atherosclerosis

Atherosclerosis is a complex phenomenon that involves endothelial dysfunction, LDL accumulation and inflammation [82,150,151,170,183]. Moreover, it is a multigenic disease and patterns may vary in lesions from different persons and at different sites of the arterial tree, suggesting genetic differences in susceptibility as well as in response to therapy. Molecular imaging has greatly increased the possibility to visualize complex biochemical phenomena underlying atherosclerosis, using highly specialized instruments in combination with targeted imaging agents. Non-invasive imaging approaches for the detection of atherosclerotic plaques that are prone to rupture would have a significant clinical impact for diagnostic and therapeutic purposes. Animal models of plaque rupture are essential for testing new imaging modalities to enable the diagnosis of the patient at risk and the design of new preventive treatments. The advances of preclinical imaging and of nanotechnologies have provided new tools to study molecular structures, cellular behaviors, and metabolic pathways that underlie atherosclerosis. In particular, the development of a wide range of imaging contrast agents, functionalized with targeting ligands such as antibodies, peptides or aptamers, could be promising for probing the molecular biomarkers of the atherosclerotic processes, promoting the translational potential of novel technologies.

Molecular imaging relies on diverse imaging techniques, which include contrast-enhanced ultrasound (CEUS), magnetic resonance imaging (MRI), positron emission tomography (PET), single-photon emission computed tomography (SPECT), X-ray computed tomography (CT), fluorescent molecular tomography (FMT) and photoacoustic imaging (PAI). Each modality presents advantages and weaknesses; therefore, multimodality imaging approaches are desirable both in preclinical research and for human applications. Importantly, longitudinal imaging studies allow for the improvement of the accuracy of the experimental results and reduce the number of animals required for experimentation, according to the 3Rs principles (Replacement, Reduction and Refinement). Table 2 compares the main characteristics, advantages and limitations of different imaging modalities with respect to atherosclerosis mouse models. A growing range of potential imaging targets have been investigated in genetically engineered mouse models, mainly ApoE^−/−^ mice, while there are few imaging studies to date that use the ApoE^−/−^C1039G^+/−^ model. In the following paragraphs of this review, the published literature on the molecular imaging of advanced atherosclerosis in murine models and its future directions will be summarized, emphasizing those strategies that provide new insight into the identification of vulnerable plaques and that have the greatest potential for clinical applications.

Table 3 describes the major targets for molecular imaging of atherosclerosis that have recently been evaluated in mouse models of vulnerable plaques, including the biological processes investigated, imaging modalities, and contrast agents used.

#### 2.3.1. Contrast-Enhanced Ultrasound Imaging

**Figure 4:**
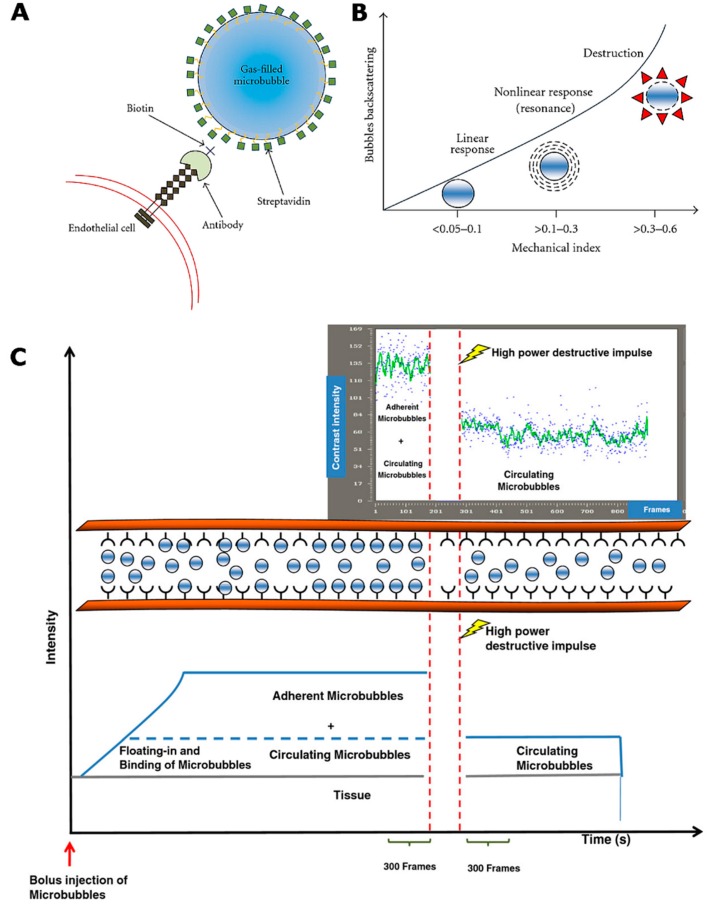
Molecularly targeted microbubbles. (**A**) Selective binding to sites of molecular expression on the endothelium: streptavidine is used for attachment of biotinylated ligands onto the shell of ultrasound contrast microbubbles; (**B**) At very low acoustic power, microbubbles oscillate in relatively symmetrical order, backscattering at the same frequency of incident ultrasound. At a slightly higher mechanical index, microbubbles oscillate in a nonlinear manner (nonlinear response), backscattering a variety of frequencies (harmonic). Higher acoustic pressures destroy the microbubbles with a high-intensity backscatter response; (**C**) Time course analysis of signal intensity before and after high-power destructive pulse and diagram representation of destructive methodology. After intravenous administration, targeted microbubbles can bind to specific antigens expressed on endothelial cells of tumor vessels (orange), whereas others remain in the vessel lumen, freely circulating. After a high-power destructive pulse (red dotted lines), both bound and circulating microbubbles are destroyed, following circulating microbubbles that arrive from outside of the scan plane, which remain freely circulating for several seconds. Contrast intensity is the sum of the intensity from the tissue, intensity from the circulating microbubbles and intensity from the microbubbles bound to receptors on endothelial cells. After digital subtraction of the video intensity calculated on 300 predestruction frames from video intensity calculated on 300 postdestruction frames, the resulting video intensity is due only to bound microbubbles. (Reprinted from Reference [207]. Copyright with permission from © 2012, Hindawi Publishing Corporation, Creative Commons Attribution License CC BY 3.0; and from Reference [208], Copyright with permission from © 2013, BioMed Central, Creative Commons Attribution License CC BY 4.0.)

Contrast-enhanced ultrasound (CEUS) is an increasingly used molecular imaging modality for the phenotypic analysis of the cardiovascular system in mouse models. In particular, ultrasound characterization of atherosclerotic plaques may be improved using contrast agents producing intense acoustic reflection and enhancing the reflection signal-to-noise ratio for blood because of their size (equal in size or smaller than red blood cells). The ultrasound contrast agents are gas-filled microbubbles, typically ranging from 1 to 5 µm in diameter, encapsulated with protein, lipid or bio-compatible polymers. Microbubbles (MB) conjugated, directly or through an avidin/streptavidin biotinylation method, with targeting ligands such as antibodies, peptides or aptamers can be used to target specific biomarkers on the endothelium or activated platelets. They are strictly intravascular agents. They act as blood pool agents and can be targeted towards endothelial cell receptors, blood cell markers or blood proteins. When acoustic waves encounter microbubbles, they alternately exert their compression with positive pressure and expansion with negative pressure. When the transmitted acoustic pressure increases, the microbubbles are compressed in a different manner, and then they expand. As a result, an asymmetric oscillation of microbubbles occurs, called “non-linear oscillation.” In non-linear oscillation, the microbubbles, instead of producing sinusoidal echoes with a frequency spectrum similar to that of the incident ultrasounds, produce, differently from the tissues, asymmetric echoes. This asymmetry produces harmonics that are utilized to differentiate the ultrasound signal from the contrast agent from that coming from the tissue. The CEUS technique cancels the linear ultrasounds from tissues and utilizes the non-linear ones from the microbubbles to form images (as shown in Figure 4) [207,208].

Moreover, microbubbles can be destroyed by an ultrasonic pulse, confirming the specificity of binding and enabling localized drug delivery for therapeutic purposes [209,210].

Contrast-enhanced ultrasound offers significant advantages, such as high spatial resolution, high sensitivity and the lack of ionizing radiation; from a perspective of clinical translation, the portability and speed of the imaging acquisition ensure that this technique is well-suited to its potential application for screening large populations and making timely diagnoses. As a limitation, the current targeted microbubble agents can only be directed against intravascular endothelial or blood cell events, reducing the potential molecular targets. Moreover, microbubbles need a critical threshold amount of molecular expression to begin to see attachment [211].

**Figure 5:**
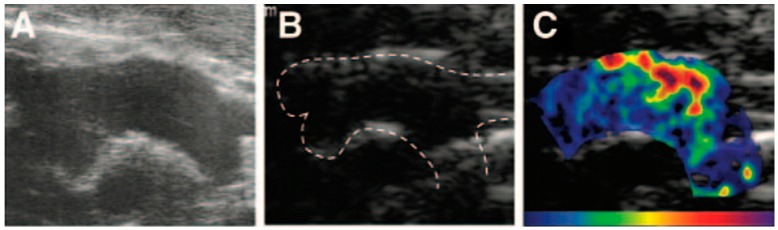
Illustration of spatial matching between morphology and targeted signal enhancement. (**A**) High-frequency ultrasound (40 MHz) image at the level of the aortic arch in a 10-week-old DKO animal; (**B**) Lower frequency multipulse contrast-specific imaging of the aorta at baseline, with the aorta defined by dashed lines, before contrast administration and (**C**) 10 min after administration of P-selectin–targeted microbubbles after background subtraction and color-coding (color scale at bottom). (Reprinted from Reference [220]. Copyright with permission from © 2010, Wolters Kluwer Health.)

Targeted contrast-enhanced ultrasonography has been shown to be a promising noninvasive imaging technique for evaluating the degree of atherosclerosis in mice [212,213,214] and may potentially be translated to clinical imaging in the future [215,216]. A number of specific targeted ultrasound probes has been tested in vivo for assessing biomarkers such as VCAM-1 [217,218,219], P-selectin [220] and von Willebrand factor [221,222] in genetically modified mouse models of atherosclerosis (as shown in Figure 5).

A thinning of the fibrous cap can ultimately induce plaque rupture and expose the core of the lesion to circulating coagulation factors. This critical event can trigger obstructive thrombosis that can cause clinical events.Molecular imaging that targets activated platelets or different steps of the coagulation cascade could be an interesting tool to detect the subocclusive thrombi and intraplaque hemorrhages that characterize vulnerable plaques. Microbubbles have been conjugated with the recombinant fusion soluble glycoprotein (GP) VI, which binds with high affinity to atherosclerotic lesions in ApoE^−/−^ mice. GPVI is a platelet-specific collagen receptor that favors platelet adhesion, activation and secretion, regulating mechanisms of atheroprogression and atherothrombosis. The GPVI receptor has high affinity for collagen within atherosclerotic plaques. The soluble GPVI receptor competes for collagen binding sites with the surface-associated platelet GPVI receptor and thereby inhibits platelet adhesion onto collagen in vitro and in vivo [223,224]. Molecular imaging signals of GPVI-targeted microbubbles were substantially enhanced in the aortic arch and in the truncus brachiocephalicus in ApoE^−/−^ compared to wild-type mice. Moreover, high-frequency ultrasound (HFU)-guided disruption of GPVI-targeted microbubbles accumulated in the atherosclerotic lesions may interfere with atheroprogression and may be an innovative therapeutic approach to prevent progression of atherosclerotic disease [202].

The glycoprotein (GP) IIb/IIIa complex, also known as αIIbβ3 integrin, is the major receptor expressed on the surface of activated platelets and is essential for their interactions with other activated platelets and adjacent cells in atherothrombosis [225,226,227]. Thus, the GP IIb/IIIa receptor is a potential marker for imaging aggregated platelets in atherosclerotic plaques. Peptides containing the Arg-Gly-Asp (RGD) sequence are highly adhesive for GP IIb/IIIa. Cyclic (c) RGD has 30-fold greater affinity for the GP IIb/IIIa complex than the linear form, which is particularly advantageous for binding under the conditions of rapid blood flow that occur in atherosclerotic arteries. A cRGD-modified MB (MB-cRGD) has recently been developed that is capable of binding to GP IIb/IIIa on activated platelets and thrombi in large arteries. It is used to examine whether GP IIb/IIIa receptors on activated platelets that are adhered and aggregated on the endothelium can serve as biomarkers of atherosclerotic plaque instability, and whether they can be identified and quantified by contrast-enhanced ultrasound using cRGD-targeted microbubbles [201].

#### 2.3.2. Contrast-Enhanced Magnetic Resonance Imaging

Contrast-enhanced MRI is a noninvasive imaging modality for characterizing the composition of atherosclerotic plaques at the molecular level, with high spatial resolution, average sensitivity and non-ionizing radiation. Innovative MRI contrast agents seem promising for discriminating between stable and unstable lesions, due to their labeling with a molecular moiety that targets pathological over-expressed hallmarks. Targeted MRI imaging probes are mostly gadolinium compounds of small molecular weight, as well as iron oxide nanoparticles. In particular, ultrasmall superparamagnetic iron oxide nanoparticles (USPIO, <60 nm) are currently widely used as contrast agents for molecular and cellular imaging, and they consist of an iron-oxide core and a shell of dextran or polymers. Gadolinium chelate (Gd-DTPA) contrast agents strongly enhance the signal profile of the atherosclerotic wall in T1-weighted sequences. Targeted probes conjugated with USPIO generally result in a marked loss of signal intensity in the atherosclerotic plaques of mouse models with a T2-weighted, rapid-acquisition, relaxation-enhanced (RARE) imaging protocol, or in a positive contrast with the “susceptibility gradient mapping” post-processing method.

Several attractive biomarkers of vulnerable atherosclerotic plaques have been evaluated using molecular MRI in the ApoE^−/−^ model, including α_v_β_3_ integrin with an RGD peptide [228] and phosphatidylserine using a peptide mimicking the endogenous ligand annexin V specific for apoptosis that was grafted to Gd-DTPA [229], or VCAM-1 and apoptotic cells using targeted peptides conjugated with USPIO [230,231]. Intraplaque fibrin plays an important role in the progression of late-stage atherosclerotic plaques. Fibrin and fibronectin accumulation in atherosclerotic plaques is associated with plaque burden; therefore, molecular imaging of these biomarkers could have great potential to prevent acute cardiovascular complications. Makowsky et al. [232] assessed intraplaque fibrin in ApoE^−/−^ mice fed a high-fat diet for one to three months using a gadolinium-conjugated agent. Molecular MRI revealed a significant increase in contrast agent uptake in brachiocephalic artery plaques, in agreement with immunohistochemical findings. Similarly, Wu et al. [233] tested the peptide CLT1, which is specific to fibrin-fibronectin complexes, conjugated with four DOTA-Gd chelates, for monitoring clots present in atherosclerotic plaques of ApoE^−/−^ mice over time. These authors found a stronger enhancement in the aortic lesions of the ApoE^−/−^ mice than in the controls at all time points and a good correlation of MRI signal progression with fibrin-fibronectin immunochemical analysis.

Distinguishing between stable and unstable rupture-prone lesions using noninvasive methods is one of the major diagnostic challenges, and the abundant presence of macrophages is a relevant imaging target for vulnerable plaque detection. Segers et al. [234] investigated the use of USPIO conjugated to a peptidic ligand of scavenger receptor AI that is highly expressed by lesional macrophages. These targeted USPIOs for inflammatory plaques have shown a significant accumulation in advanced atherosclerotic plaques of ApoE^−/−^ mice. Another recent study of Parolini et al. [235] evaluated the potential of a new blood-pool contrast agent referred to as B22956/1 to identify vulnerable plaques in the brachiocephalic arteries of ApoE^−/−^ mice. These authors highlighted a significant correlation between MRI signal enhancement and macrophage content in atherosclerotic lesions.

**Figure 6:**
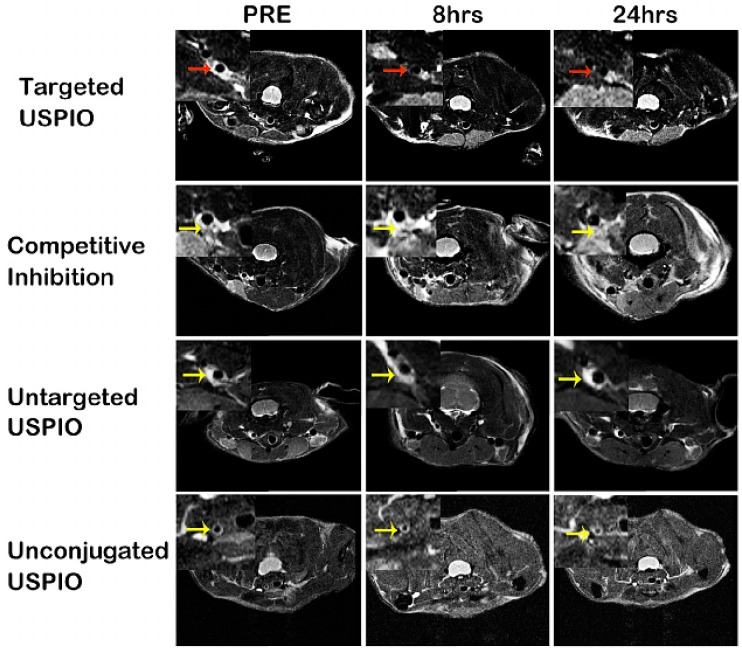
USPIOs MRI of ApoE^−/−^ mice in vivo. Representative in in vivo carotid atherosclerotic lesion in ApoE^−/−^ mice pre-, 8 and 24 hrs after administration of various USPIOs. The red arrows indicate the location of signal loss within the plaque while the yellow arrows indicate the location of carotid atherosclerotic lesions with limited rSI changes. (Reprinted from Reference [237], Crown copyright with permission from © 2014, Elsevier Ltd.)

Tarin et al. [236] also tested a targeted probe based on gold-coated iron oxide nanoparticles vectorized with a specific antibody for the CD163 receptor, highly expressed on macrophages at inflammatory sites, in ApoE^−/−^ mice. A significant signal variation over time was observed in the aortic walls of ApoE^−/−^ mice with respect to the pre-injection signal or nontargeted nanoparticles; the MRI results were confirmed in autoptic samples by specific immunostaining. Lectin-like Oxidized Low-Density Lipoprotein Receptor 1 (LOX-1) is a membrane receptor expressed on endothelium, inflammatory cells and SMCs present in atherosclerotic plaques which plays a crucial role in the destabilization and rupture of lesions. Wen et al. [237] reported a sensitive and specific LOX-1–targeted USPIO, producing a significant signal loss in MR images at the level of atherosclerotic lesions in carotid arteries of ApoE^−/−^ mice (as shown in Figure 6).

The MRI is characterized by high spatial resolution; nevertheless, it has relatively low sensitivity in comparison with other imaging techniques such as nuclear medicine or optical imaging. In particular, near-infrared fluorescence (NIRF) imaging can overcome this limitation, due to its high sensitivity, and also appears to be easy to perform and of relatively low cost. Therefore, several previously published studies have proposed interesting bimodality imaging strategies with in vivo and ex vivo NIRF: optical imaging was used to confirm nanoparticle uptake in the lesions, and MRI helps to accurately determine the location of in vivo fluorescence signals. Van Tiborg et al. [198] characterized a micellar nanoparticle conjugated to annexin A5, carrying both Gd-labeled lipids and a fluorescent dye. In vivo MR imaging highlighted cells, exposing phosphatidylserine in atherosclerotic lesions of ApoE^−/−^ mice, and ex vivo NIR fluorescence imaging of excised aortas was used to validate the quantification of nanoparticles in the plaques.

Very recently, Wang et al. [238] illustrated a novel multi-modality molecular imaging probe obtained by conjugating polyclonal profilin-1 antibody and NHS-Cy5.5 fluorescent dye to the surface of DMSA-Fe_3_O_4_ nanoparticles (PC-NPs) that can be used to perform noninvasive imaging of atherosclerotic plaques at the level of the carotid artery in ApoE^−/−^ mice. Profilin-1 is an actin-binding protein involved in the modulation of cytoskeleton architecture that is over-expressed in activated vascular SMCs present in atherosclerotic plaques, and therefore it is recognized as a promising potential target to comprehensively assess the vulnerability of plaques. To date, any other molecular probe targeted at activated SMCs in plaques is described. These authors revealed the accumulation of PC-NPs in atherosclerotic plaques of the analyzed model using in vivo MRI and NIRF imaging: fluorescence signals in the carotid arteries were significantly higher in ApoE^−/−^ mice compared to controls and significant T2-weighted MRI signal attenuation was observed, with a high correlation between the fluorescence imaging intensity and MRI signal changes. Moreover, this targeted probe has also been shown to be helpful in evaluating the therapeutic efficacy of atorvastatin through dynamic monitoring [238].

#### 2.3.3. Nuclear Imaging Techniques and X-ray Computed Tomography

Nuclear techniques such as PET and SPECT potentially provide detection sensitivities in the nanomolar-picomolar range. Such functional imaging enables the investigation of biological events that lead to plaque rupture with high specificity and offers relevant potential results of clinical translatability from basic research to identify high-risk patients. Furthermore, the combination of the nuclear medicine images with the morphological information provided by CT in hybrid scanners, or with the high soft tissue contrast obtained through MRI, has the potential to map molecular signals with precise anatomic details. Also, more recently, the association with optical imaging was shown to be particularly helpful for testing innovative probes and for in vivo tracking of targeted cells.

Macrophage markers, such as CD68 glycoprotein, which binds to low-density lipoprotein, correlate well with atherosclerotic lesion vulnerability. Nahrendorf et al. [239] described a novel trimodal nanoparticle, which targets the CD68 macrophage marker, consisting of an iron oxide core for MRI and an optically detectable near infrared fluorochrome that is radiolabeled with the PET tracer ^64^Cu. This agent was found to accumulate predominantly in macrophages present in plaques at the level of the carotid artery and aorta of ApoE^−/−^ mice, as confirmed by ex vivo autoradiography and fluorescence microscopy. More recently, Seo et al. [240] performed in vivo PET-CT studies in atherosclerotic ApoE^−/−^ mice using a fluorine-18–labeled dendritic form of a cyclic nine-amino-acid peptide, named LyP-1, which targets p32 proteins on macrophages and which was labeled with fluorine-18. PET-CT co-registered images have shown significant uptake of the radiotracer in the aortic root and descending aorta after two hours of biodistribution.

Macrophages can also be targeted with antibodies against LOX-1, which is upregulated in response to high levels of oxidized LDL and proinflammatory cytokines. For imaging purposes, Li et al. [241] conjugated a LOX-1 antibody with the SPECT radionuclide ^99^mTechnetium (99mTc), showing that this probe was able to reliably target macrophages in vivo in ApoE^−/−^ mice and LDLr^−/−^ (LOX1^−/−^) mice. The peripheral benzodiazepine receptor (PBR), also known as translocator protein (TSPO), is minimally expressed in non-inflamed tissue and is highly expressed on activated macrophages; therefore, it is exploited as a molecular imaging target. 

**Figure 7:**
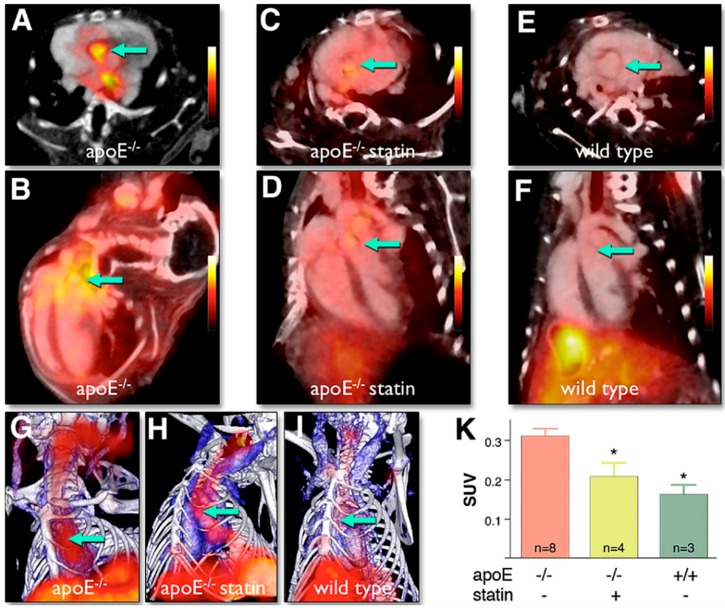
PET-CT in ApoE−/− and statin-treated mice. PET-CT imaging shows uptake of ^18^F-4V in the aortic root (arrows) and arch of atherosclerotic mice. Uptake is lower in statin-treated and in wild-type mice. (**A**,**C**,**E**) Short-axis views; (**B**,**D**,**F**) Long-axis views; (**G**,**H**,**I**) Three-dimensional maximum intensity projection. Bone is shown in white, vasculature in blue, and ^18^F-4V PET signal in red. The PET signal occurs in the carotid arteries, and background signal in the liver, in addition to the strong uptake of ^18^F-4V PET observed in the root and arch (arrow). *K* = quantification of PET signal as the standard uptake value (SUV). Mean ± SEM, * *p* < 0.05. Abbreviations as in Figure 1, Figure 2 and Figure 5. (Reprinted from Reference [243]. Copyright with permission from © 2009, American College of Cardiology Foundation, Elsevier Inc.)

Foss et al. [242] developed a SPECT imaging agent, [^125^I]iodo-DPA-713, which targets macrophages, to selectively detect macrophage infiltrates along the descending aorta and within the myocardium of the ApoE^−/−^ mice. Radiotracers targeting VCAM-1 have been validated in murine experimental systems as attractive tools for imaging vulnerable plaques. Atherosclerotic lesions in the aortic arches of ApoE^−/−^ mice have been successfully identified by Nahrendorf et al. [243] using the peptide 4V, and very recently by Bala et al. [244] through the specific nanobody cAbVCAM-1-5, both radiolabeled with fluorine-18, showing a significant correlation between the uptake of the radiotracers and the level of expression of the VCAM-1 receptor in atherosclerotic lesions assessed by ex vivo analyses (as shown in Figure 7).

Similarly, Broisat et al. [245] tested anti–VCAM-1 nanobodies labeled with ^99^mTc both in vitro on murine and human endothelial cells and in vivo at the level of the aortic arch in ApoE^−/−^ mice. Dimastromatteo et al. [246] demonstrated in vivo the uptake of the peptide 99mTc-B2702p1 at the level of the carotid arteries in the same model, confirming VCAM-1 expression using immunohistology.

Other relevant targets of plaque vulnerability, investigated through nuclear medicine imaging in the ApoE^−/−^ murine model, include other biomarkers of inflammation such as the following: P-selectin [247,248]; α_v_β_3_ receptor with an RGD-galacto peptide [249] or the novel integrin ligand flotegatide [250], both labeled with fluorine-18; phosphatidylserine using ^99^mTc-annexin V [197] or ^99^mTc-AnxF568 and ^124^I-Hypericin [251] for apoptosis imaging; and extracellular matrix proteins such as GPVI with a ^64^Cu-labeled GPVI fragment crystallized [252] or fibronectin using a specific ^99^mTc-antibody [253]. Finally, in an attempt to improve the detection of vulnerable lesions, contrast agents for CT imaging of macrophage-rich atherosclerotic plaques have been developed and tested in the major mouse models of atherosclerosis, for example liposomal-iodine [254] or PEGylated gold nanoparticles [255] in ApoE^−/−^ mice and, more recently, gold particles of 15 nm with a polyethylene glycol coating called Aurovist in the ApoE^−/−^C1039G^+/−^ model [43].

#### 2.3.4. Targeted Fluorescence Imaging

Currently, fluorescence imaging is emerging in preclinical research because it offers several advantages: high sensitivity, the possibility of simultaneous multi-spectral imaging, high-throughput capabilities, cost-effectiveness, and the absence of ionizing radiation. This technique can be applied both ex vivo for immunofluorescence microscopy and for in vivo applications such as intravital microscopy or tomographic imaging. In particular, FMT in the NIR window is gaining great relevance in cardiovascular research for longitudinal studies in mouse models of atherosclerosis. Atherosclerosis research using FMT is still sparse, but the previously mentioned peculiarities make it an attractive in vivo alternative to nuclear imaging. FMT is based on the collection of photons propagated in deep tissues from different points of view to obtain the tomographic distribution of fluorochromes.

A number of fluorescent targeted probes are available, with the possibility of obtaining in vivo quantitative three-dimensional data of fluorescence signal distribution. These probes are excitable with a laser at the appropriate wavelength, and, after excitation, they emit light at a higher wavelength, detectable with appropriate charge-coupled device cameras. FMT offers an improved penetration depth of light in the near-infrared spectrum and limited autofluorescence but has limited spatial resolution and is currently restricted to preclinical field or ex vivo human samples [256]. Moreover, multimodality imaging with the association of FMT-CT or -MRI holds promise for noninvasive imaging of murine models of atherosclerosis, adding anatomical details to molecular signals [257,258,259]. Macrophage content correlates with plaque vulnerability because the fibrotic cap can be destabilized by the secretion of matrix-degrading enzymes, contributing to acute thrombotic complications. 

**Figure 8:**
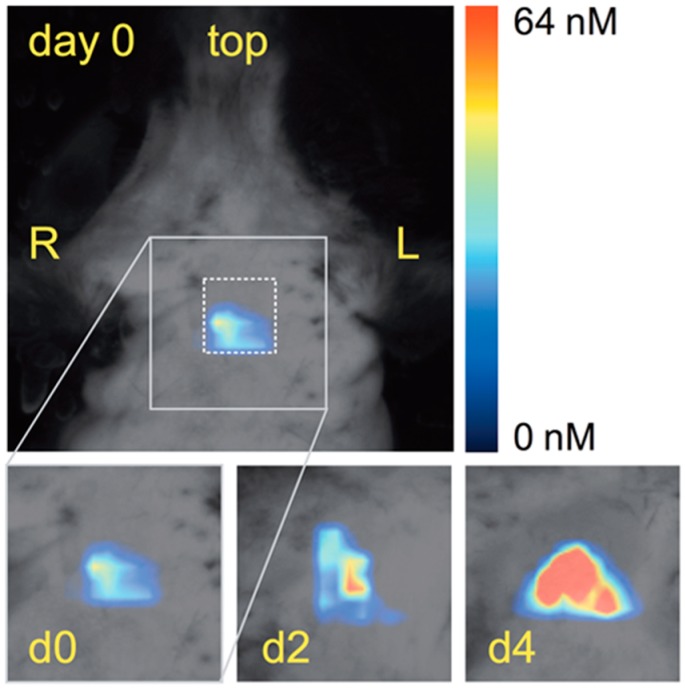
Fluorescence-mediated tomography (FMT) reliably quantifies 1,1′-dioctadecyl-3,3,3′,3′-tetramethylindotricarbocyanineiodide (DiR)-enhanced green fluorescent protein (eGFP) macrophage recruitment over time in a region of interest covering aortic sinus, aortic arch, and brachiocephalic artery of ApoE^−/−^ mice. (Reprinted from Reference [261]. Copyright with permission from © 2013, Wolters Kluwer Health.)

Cathepsins and metalloproteinases are highly expressed in rupture-prone plaques, and the activity of these macrophage-related molecules has been evaluated at the level of the ApoE^−/−^ mouse aortas both in vivo and ex vivo with fluorescent imaging [260]. Nahrendorf et al. [257] have also monitored the macrophage-protease function in vivo by FMT as a biomarker of destabilized atheromata and have coregistered FMT datasets with high-resolution CT angiography, localizing the highest fluorescence signal in the aortic root and arch of ApoE^−/−^ mice. Moreover, the same authors showed that FMT-CT may be useful for monitoring the efficacy of atorvastatin treatment in ApoE^−/−^ mice. In agreement, Larmann et al. [261] used FMT to test the plaque-stabilizing effects of high-dose atorvastatin treatment in ApoE^−/−^ mice through in vivo tracking of the recruitment of near-infrared fluorescent-labeled macrophages (as shown in Figure 8).

FMT-based quantification of macrophage recruitment in vulnerable plaques has demonstrated lesion stabilization after four days of atorvastatin therapy. Similarly, a recent study has successfully used FMT to visualize protease activity in ApoE^−/−^ mice and to assess the efficacy of an anti-inflammatory nanotherapeutic [262].

Apoptosis correlates with plaque vulnerability and, therefore, represents an important diagnostic target. Van Tiborg et al. [198] developed a fluorescent nanoparticle functionalized for annexin 5 that was applied both in vivo using MRI and ex vivo with near-infrared fluorescence imaging of excised aortas. Calcification in atherosclerotic plaques is associated with macrophage infiltration and is predictive of cardiovascular events. Plaque rupture occurs particularly at interface areas between high- and low-density tissues, and microcalcification in the fibrous cap induces microfractures. Aikawa et al. [139] used a bisphosphonate-derivatized near-infrared fluorescent agent to visualize osteogenic activity and iron oxide fluorescent nanoparticles for detection of macrophages in ApoE^−/−^ mouse aortas, demonstrating with ex vivo fluorescence imaging an increased osteogenic activity in macrophage-rich atherosclerotic plaques of 20- to 30-week-old mice.

More recently, Lin et al. [263] evaluated cathepsin activity and α_v_β_3_ expression in ApoE^−/−^ mice using FMT, demonstrating that targeted NIRF agents can be successfully employed for molecular imaging of vulnerable plaques at the level of the aortic root and arch, and the descending aorta and carotid arteries. In preclinical testing, cathepsin agents can monitor the anti-inflammatory effects of ezetimibe. Also, in this case, the FMT signal was conveniently co-registered with CT angiographic images using anatomical details from CT to further identify the location of the molecular information. Similarly, Yao et al. [264] have shown that α_v_β_3_ is highly expressed in atherosclerotic lesions of the ApoE^−/−^ model using a cyclic RGD peptide (cRGDyK) conjugated with the near-infrared dye Cy5.5 both in vivo and in excised carotid arteries. cRGDyK-Cy5.5 co-localization with MAC-3 expression was confirmed using fluorescence confocal microscopy.

#### 2.3.5. Photoacoustic Imaging

Photoacoustic imaging (PAI), also called optoacoustic tomography (OAT), is a hybrid imaging modality that uses nonionizing optical radiation and ultrasonic detection. Recent advances in laser technology and detection strategies have led to significant improvements in the capabilities of optoacoustic systems. PAI is showing real promise as a convenient alternative to other imaging modalities. PAI combines the advantages of both optical and ultrasonic imaging methods, offers higher spatial resolution and allows imaging of deeper tissues compared to optical imaging techniques [265,266]. PAI is based on the photoacoustic effect: a short-pulsed laser irradiates biological tissues and induces ultrasonic waves due to transient thermoelastic expansion [265]. In comparison to optical imaging, PAI allows high-resolution visualization (≤100 μm) with a penetration depth within tissues of several centimeters, because it is less influenced by photon scattering.

Applications in the field of molecular imaging provide functional information regarding cellular events by using endogenous chromophores (hemoglobin, melanin, and lipids) and a great variety of exogenous contrast agents [267,268,269]. Furthermore, OAT contrast agents can be combined with CEUS for advantageous dual modality imaging approaches [270,271]. PAI has been used for various biological applications such as angiogenesis, oxygen saturation and drug response for oncologic screening and studies on brain functions, including preclinical research using small animal models of atherosclerosis [272,273]. Despite its attractive features, PAI experienced a slow development and is still facing some issues for routine adoption, relying on the development and validation of molecular agents tailored to this imaging modality. Few studies have explored the feasibility and potential of this technique in atherosclerosis research. The main strategies explored through in vitro or ex vivo PA studies have included targeting of foamy macrophages and of inflammatory molecular biomarkers such as ICAM-1, VCAM-1 and E-selectin using gold nanoparticles (AuNPs) [274,275,276].

**Figure 9:**
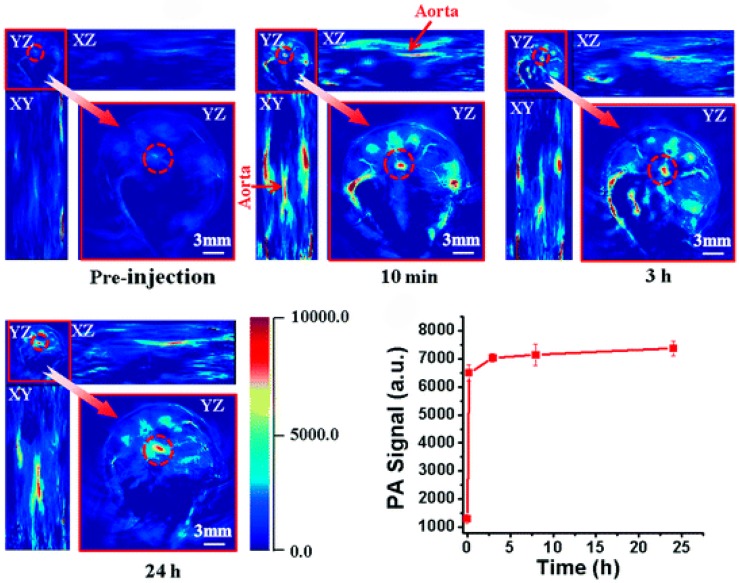
Representative PA imaging of abdominal aorta in an ApoE^−/−^ mouse after intravenous injection of ICG@PEG-Ag2S (longitudinal and transverse view): a low contrast in the whole body of the mouse is evident, while a remarkable enhancement of the PA intensity in the region of the aorta (as indicated by red arrows and red circles) was observed over time. Graph shows quantitative changes over time of PA signals in the aorta of the mouse corresponding to the red circle in images. (Reprinted from Reference [278]. Copyright with permission from © 2016, Royal Society of Chemistry.)

Very recently, some pilot preclinical studies have been conducted in ApoE^−/−^ mice to test the in vivo potential of molecular PA contrast agents to identify stable versus vulnerable plaques such as a VCAM-1–targeting gold nanoshell probe, which has highlighted a higher PA signal in the aortic arches of ApoE^−/−^ mice fed a WTD compared to controls [277] and indocyanine green (ICG) conjugated with a PEGylated polymer in a new ICG@PEG-Ag2S nanoprobe that was shown to selectively accumulate in the atherosclerotic plaques due to its lipophilicity and produced a six-fold enhancement of the PA signal intensity in the region of the aorta 3 h post-injection in ApoE^−/−^ mice compared to the signal produced after administration of free ICG [278] (as shown in Figure 9).

## 3. Conclusions and Future Directions

Atherosclerosis can start in the late years of childhood and can remain silent for many years. It becomes symptomatic when it interferes with the circulation and blood supply, causing heart attack, stroke, or ischemic symptoms. However, this mechanism is responsible for acute events in only 30%–40% of patients. A significant number of people with myocardial ischemia do not have plaques that significantly narrow the arteries. A total of 60%–70% of patients with acute coronary syndrome or sudden cardiac death have thrombosis associated with rupture-prone plaques that have been shown in post-mortem evaluation to have specific characteristics such as increased macrophage activity and a peculiar molecular pattern. These features appear in an atherosclerotic plaque prior to rupture.

As outlined in the “response-to-injury” hypothesis by Ross et al. [150], the endothelial denudation injury or activation was considered the first step in the development of atherosclerosis. The monocyte-endothelial interactions giving rise to foam cells and growth factor–induced SMC proliferation trigger lesion formation. Presently, the knowledge of the atherogenic process has been considerably refined using murine models of atherosclerosis through recognition of other important factors such as the subendothelial accumulation of apolipoprotein B–containing lipoproteins; the roles of macrophages, neutrophils, T cells, and dendritic cells; and the identification of specific chemotactic signals that regulate the recruitment of inflammatory cells into the lesion, such as extracellular RNA (eRNA) acting as a cofactor of VEGFR-2–coupled or other intracellular signaling pathways [279,280]. The existence of a pathological interface between lipids and inflammation, the role of cytokines and the importance of highly specific molecular targets that can be used to develop diagnostic imaging systems and innovative therapeutic tools, such as RNA-degrading enzymes, RNA interference or silencing systems [279], are made quite clear by these studies. Thus, there is an urgent need for identifying vulnerable atherosclerotic plaques before acute cardiovascular events.

Many of the currently available clinical diagnostic methods still provide minimal information about the biological characteristics of plaques and/or are invasive. The imaging methods currently used provide only partial quantitative and morphological data on the atherosclerotic plaque. The cellular and molecular processes that characterize the atherosclerotic plaques require more advanced methods that can best represent the peculiarities of the pathological events with respect to size. The development of molecular imaging using probes for specific biological functions could be of great value to study the evolution and the chemical outcome of the atherosclerotic plaque and for the prediction of vulnerability. The study of atherosclerosis in animal models is of great relevance for the advancement of knowledge, but some precautions are required when relevant data are extrapolated from animals for new diagnostic and therapeutic tools. Proper design and generation of animal models are essential to make preclinical data more reproducible and translatable to the clinic. Atherosclerosis is a complex disease. The animal models are a simple representation of this complex system. Therefore, a single animal model may not be able to reproduce all aspects and all stages of the atherosclerotic process in humans in all of its complexities but rather may represent a specific aspect of the disease. In atherosclerosis research, mice are nowadays the most commonly used animals. Generally, the lipid metabolism in wild-type mice is significantly different from humans. The major fraction of plasmatic lipoproteins in wild-type mice is HDL, which is different from humans where LDL or VLDL lipoproteins are mainly represented, and therefore mice are well protected from atherosclerosis due to a beneficial cholesterol spectrum in the blood. Nevertheless, the C57BL6 strain of inbred mice is relatively susceptible to atherosclerosis, and by feeding them with an atherogenic diet, development of atherosclerosis can be augmented to such an extent that the resulting advanced lesions are useful to study disease mechanisms. Overcoming the limits of this latter model, the advent of the genetically modified mice has revolutionized the study of the pathogenetic mechanisms of atherosclerosis.

In particular, ApoE^−/−^ mice are currently the most well-characterized mouse model of atherosclerosis used by research groups worldwide. However, the ApoE^−/−^ mouse is a model of a rare disease in humans, named homozygous familial hypercholesterolemia, while the atherosclerosis induced by LDL accumulation is the most common pathological mechanism in humans. Additionally, several systemic factors could modify the course of the disease in the animal models, such as plasmatic levels of HDL, insulin resistance, and hemodynamic factors, which could make the process more or less different from that of humans. Moreover, considering that atherosclerosis development is largely dependent on local inflammation, it is important to consider that mechanisms of inflammation in atherosclerosis could differ between mice and humans [281]. Furthermore, the choice of the lesional area of the disease may affect the usefulness of information obtained from the animal models and is essential to bridge the translational gap between preclinical and clinical research. Most preclinical studies refer to the aortic root, the whole descending aortic surface or the innominate artery. This endpoint is relevant for the early stages of the disease but is poorly predictive of the clinical features of the human condition.

Very recently, the ApoE^−/−^Fbn1C1039G^+/−^ mouse model has been developed. These mice exhibit vulnerable atherosclerotic plaque phenotypes, with a greater incidence of plaque rupture in comparison to the ApoE^−/−^ or LDL^−/−^ mice, which exhibit mainly stable plaques.

The new generation of imaging modalities comprises promising tools for the prediction of vulnerable atherosclerotic plaques. Although novel molecular imaging approaches for atherosclerosis have been tested in the previously mentioned experimental systems, accurate assessment of plaque characteristics and vulnerability is still in its infancy. Each imaging technique has its own strengths and drawbacks. At the moment, CEUS and molecular MRI appear to be the most suitable and promising because they have been applied to research the analysis of the compositional features of vulnerable plaques, they show high resolution and sensitivity, they use nonionizing radiation and they have potential clinical utility. The main concerns to overcome are: improvement of the sensitivity and specificity of the molecular probes; complex extrapolation of results from animal models; and scientific as well as financial challenges for translating new molecular imaging ligands from preclinical research to clinical practice. Further investigations of the mechanisms of plaque destabilization and rupture and technological advances of imaging equipment are required to reduce both mortality and morbidity worldwide. Successful translation of targets and ligands to clinical molecular imaging of the vulnerable plaque may also offer novel therapeutic perspectives for vulnerable plaques such as targeted drug delivery.

## Figures and Tables

**Table 1:** Summary of major mouse models of atherosclerosis with features of vulnerability (highlighted in blue).

Model	Mouse Strain	Stage of Lesion Formed (I–VI)	Lesion Characteristics	Area of Lesion Characterization	Modified Diet Required	Commercial Availability of Strain
Diet-induced	C57Bl/6J	I	Predominantly lipid-laden foam cells	Aortic root, aorta	Yes	Jackson ,Taconic, Charles River
Genetically modified	LDL^−/−^ [64]	I–IV	Progression from predominantly lipid-laden foam cells to lesions with necrotic core and fibrous caps	Aortic root, aorta	Yes	Jackson
Genetically modified	ApoE^−/−^ [56,57]	I–V	Progression from predominantly lipid-laden foam cells to lesions with necrotic core and fibrous caps	Aortic root, aorta; rare vulnerable plaques in innominate artery	No; fat diet accellerates atherogenesis	Jackson
Genetically modified	ApoE^−/−^ Fbn1(C1039G)^+/−^ [142]	I–VI	Development of vulnerable plaques prone to rupture; acute cardiovascular events (stroke, miocardial infarction)	Aortic root, aorta, innominate artery	No; fat diet accellerates atherogenesis	Jackson for ApoE^−/−^ and Fbn1(C1039G^+/−^) breeders
Genetically modified	Apo E*3-Leiden [73]	I–IV	Progression from predominantly lipid-laden foam cells to lesions with necrotic core and fibrous caps	Aortic root, aorta, common carotid arteries	No; fat diet accellerates atherogenesis	Jackson
Collar-induced	ApoE^−/−^	I–VI	Development of vulnerable plaques for biomechanical alterations	Common carotid arteries	No; fat diet accellerates atherogenesis	Jackson
Tandem stenosis	ApoE^−/−^	I–VI	Development of vulnerable plaques for biomechanical alterations	Common carotid arteries	No; fat diet accellerates atherogenesis	Jackson
Collar + Ad-p53 + phenylephrine- induced	ApoE^−/−^	I–VI	Development of vulnerable plaques for biomechanical alterations, apoptosis of SMCs and hypertension	Common carotid arteries	No; fat diet accellerates atherogenesis	Jackson
Ligation plus cuff	ApoE^−/−^	I–VI	Development of vulnerable plaques for biomechanical alterations	Common carotid arteries	No; fat diet accellerates atherogenesis	Jackson
Partial ligation of carotid and renal arteries	ApoE^−/−^	I–VI	Development of vulnerable plaques for biomechanical alterations and hypertension	Common carotid arteries, renal arteries	No; fat diet accellerates atherogenesis	Jackson
Angiotensin II	ApoE^−/−^	I–VI	Development of vulnerable plaques with neovascularization, hemorragies and inflammation	Aortic root, aorta, innominate artery	No; fat diet accellerates atherogenesis	Jackson
Adenovirus-induced gene mutation	ApoE^−/−^	I–VI	PCSK9^DY^ mutation, prothrombin overespression; fibrous cap disruption, hemorrhagies and thrombosis	Aortic root, aorta, innominate artery	No; fat diet accellerates atherogenesis	Jackson

**Table 2:** Noninvasive molecular imaging in mouse models of vulnerable atherosclerotic plaques.

Imaging Modality	Spatial Resolution	Sensitivity (mol/L)	Contrast Agent	Probe Concentration	Advantages	Limits
Ultrasound	50–500 μm	Not well characterized yet	Microbubbles	µM to nM	Real-time Low cost High temporal resolution (0.1–100 s) No ionizing radiation	Operator-dependent
Magnetic Resonance	10–100 μm	10^−3^–10^−5^	Gadolinium-based contrast agents Iron oxide and other superparamagnetic nanoparticles (USPIO, SPIO)	mM to nM	High tissue contrast and functional parameters No ionizing radiation	High cost Operator-dependent
Nuclear imaging	PET 1–2 mm		Positron or gamma ray emitting radionuclides (^18^F, ^64^Cu, ^99m^Tc tracers)	pM	Molecular and functional parameters High sensitivity	Ionizing radiation Limited spatial resolution (mm) High-medium cost
		10^−11^–10^−12^				
	SPECT 0.5–2 mm	10^−10^–10^−11^				
X-ray computed tomography	30–400 μm	10^−2^–10^−3^	Iodinated particles Gold nanorods	mM to nM	Fast acquisition time High temporal resolution (1–300 s) Provides molecular and structural information	Ionizing radiation Low soft tissue contrast resolution Medium cost
Fluorescence tomographic imaging	1–2 mm	10^−10^–10^−11^	NIR Fluorophores	nM to pM	High sensitivity No ionizing radiation Low cost	Limited depth of penetration (1–20 mm) Limited spatial resolution (mm)
Photoacoustic imaging	<100 μm	<10^−12^	NIR Fluorophores	nM to pM	High sensitivity No ionizing radiation High depth of penetration (<5 cm) Low cost	Data post-processing and acquisition procedures still being optimized

**Table 3:** Summary of the major targets for molecular imaging of atherosclerosis recently evaluated in mouse models with features of vulnerability.

Molecular Target	Biological Events	Imaging Techniques	Imaging Probes
VCAM1-R; ICAM1-R; P-selectin	Vascular inflammation	UBM, MRI, PET, SPECT, PAI	Targeted microbubbles, targeted USPIO, ^18^F-, ^99m^Tc-labeled VCAM1 antibodies, NIR Fluorophores
Phosphatidylserine	Apoptosis, vulnerable plaque, atherothrombosis	MRI, SPECT, FMT	Targeted USPIO, ^99m^Tc-labeled annexin 5 or other tracers, NIR dyes conjugated with annexin 5
α_v_β_3_	Neoangiogenesis	MRI, PET, FMT	Gadolinium-labeled RGD probes, ^18^F-labeled RGD or other tracers, NIR dyes conjugated with RGD or other probes
GPVI-R	Platelet adhesion, atherothrombosis	UBM, PET	Targeted microbubbles, ^64^Cu-labeled GPVI fragment
GP IIb/IIIa-R	Platelet adhesion, atherothrombosis	UBM	Targeted microbubbles
Fibrin-fibronectin complex	Atherothrombosis	MRI, SPECT	Gadolinium-labeled CLT1 peptide or other agents, ^99m^Tc-labeled antibodies
Von Willebrand factor	Atherothrombosis	MRI, SPECT	Targeted microbubbles,
LOX-1	Macrophagic lipid uptake	MRI, SPECT	Targeted USPIO, ^99m^Tc-labeled antibodies
TSPO	Activated macrophages	SPECT	[^125^I]iodo-DPA-713
Cathepsins and metalloproteinases	Macrophagic proteinases activity	FMT	NIR dyes
Macrophages infiltration	Macrophage-rich, rupture-prone plaques	CT, MRI, PET, FMT, PAI	Liposomal-iodine formulations, PEGylated gold nanoparticles, gold-coated iron oxide nanoparticles targeted for CD163 receptor antibody, trimodality ^64^Cu- iron oxide-NIR dye nanoparticle targeted for CD68, ^18^F-LyP-1 targeted for p32, NIR Fluorophores

## Abbreviations

AHA	American Heart Association
ApoE^−/−^	apolipoprotein-E-knockout
WTD	western type diet
Fbn1	fibrillin-1
MRI	magnetic resonance imaging
CEUS	contrast-enhanced ultrasound
microCT	micro-computed tomography
HDL	high-density lipoprotein
LDL	low-density lipoprotein
VLDL	very low-density lipoprotein
SMCs	smooth muscle cells
LDL^−/−^	low-density lipoprotein-knockout
MMP	matrix metalloproteinase
LPS	lipopolysaccharide
Ang II	angiotensin II
MCP-1	monocyte chemoattractant protein-1
DIO	diet-induced obese
DD	diabetogenic diet
STZ	streptozotocin
T1D	type 1 diabetes
hAR	human aldose reductase
T2D	type 2 diabetic
PCSK9	proprotein convertase subtilisin/kexin type 9
VCAM-1	vascular cell adhesion molecule-1
ICAM-1	intercellular adhesion molecule-1
VLA 4	very late antigen-4
TLR-4	Toll-like receptor 4
FR β	folate receptor β
CD68	Cluster of Differentiation 68
VEGF	vascular endothelial growth factor
VEGFRs	vascular endothelial growth factor receptors
VEGFR-1	vascular endothelial growth factor receptor 1
VEGFR-2	vascular endothelial growth factor receptor 2
PS	phosphatidylserine
A5	annexin V
GPVI	glycoprotein VI
PET	positron emission tomography
SPECT	single-photon emission computed tomography
CT	X-ray computed tomography
FMT	fluorescent molecular tomography
PAI	photoacoustic imaging
HFU	high-frequency ultrasound
MB	microbubbles
GP IIb/IIIa	glycoprotein (GP) IIb/IIIa complex
RGD	Arg-Gly-Asp sequence
cRGD	cyclic RGD peptide
USPIO	ultrasmall superparamagnetic iron oxide nanoparticles
Gd-DTPA	gadolinium chelates
RARE	rapid-acquisition relaxation-enhanced
LOX-1	Lectin-like Oxidized Low-Density Lipoprotein Receptor 1
NIRF	near-infrared fluorescence
PC-NPs	DMSA-Fe_3_O_4_-nanoparticles
LyP-1	cyclic 9-amino acid peptide
^99m^Tc	^99m^Technetium
PBR	peripheral benzodiazepine receptor
TSPO	translocator protein
OAT	optoacoustic tomography
AuNPs	gold nanoparticles
ICG	indocyanine green
eRNA	extracellular RNA
